# On-Site Periodontitis Diagnosis via Room-Temperature Oral Exhalation H_2_S Sensor Based on Yb-Doped Bi_2_S_3_ Nanoribbon

**DOI:** 10.1007/s40820-026-02273-x

**Published:** 2026-07-08

**Authors:** Ping Guo, Kai Wang, Xuyang An, Xuanyu Ren, Xinxin He, Yinhua Hu, Lijie Zhou, Guohua Cai, Lifeng Wang, Tiezhu Liu, Wenyu Ge, Jia Zhang

**Affiliations:** 1 State Key Laboratory of Robotics and System, Harbin Institute of Technology, Harbin, 150080 People’s Republic of China; 2 Department of Stomatology Center, Heilongjiang Provincial Hospital, Harbin, 150036 People’s Republic of China; 3 Key Laboratory of Bio-Based Material Science and Technology of the Ministry of Education, Northeast Forestry University, Harbin, 150040 People’s Republic of China; 4 School of Mechanical and Power Engineering, Harbin University of Science and Technology, Harbin, 150080 People’s Republic of China; 5 The Second Affiliated Hospital of Harbin Medical University, Harbin, 150081 People’s Republic of China; 6 School of Science, City Campus Australia, RMIT University, 124 La Trobe Street, Melbourne, VIC 3000 Australia; 7 Key Laboratory of Micro-Systems and Micro-Structures Manufacturing, Ministry of Education, Harbin Institute of Technology, Harbin, 150080 People’s Republic of China

**Keywords:** Periodontitis diagnosis, On-site, Oral exhalation, H_2_S sensor, Yb-doped Bi_2_S_3_ nanoribbon

## Abstract

H_2_S gas in oral exhalation is a critical biomarker for early periodontitis diagnosis, yet practical on-site detection via portable sensors remains challenging. Herein, we report a room-temperature H_2_S sensor based on CAU-17-derived Yb-doped Bi_2_S_3_ (Yb-Bi_2_S_3_) nanoribbons for rapid periodontitis screening. The sensor exhibits segmented linearity ranging from 5 ppb to 5 ppm, with a 3.31 response at 100 ppb, 11 s response time, and superior selectivity. Mechanism analysis combined with density functional theory calculations demonstrates that the superior detection performance originates from Yb doping-induced lattice distortion in Bi_2_S_3_, which enhances oxygen anion formation and specific H_2_S adsorption capability. Notably, the sensor quantifies H_2_S production from cultured Porphyromonas gingivalis, corroborating the correlation between oral pathogens, H_2_S, and periodontitis. Additionally, it effectively discriminates exhaled H_2_S levels on-site between healthy individuals and periodontitis patients in clinical validation. This work provides a promising platform for sensitive biomarker detection, paving the way for advanced intelligent medical diagnostics.

**Supplementary Information:**

The online version contains supplementary material available at 10.1007/s40820-026-02273-x.

## Highlights

- A novel Yb-doped Bi_2_S_3_ H_2_S sensor was fabricated for periodontitis exhalation diagnosis, which enables room temperature detection with high response (3.31/100 ppb) and superior selectivity.
- Mechanism analysis combined with density functional theory calculations confirms that Yb doping-induced lattice distortion in Bi_2_S_3_ enhances O^2-^ formation and H_2_S adsorption.
- The sensor quantifies H_2_S produced by Porphyromonas gingivalis to confirm the association among pathogens, H_2_S and periodontitis, and is integrated into a portable analyzer for rapid, real‑time diagnosis and severity assessment of periodontitis clinically.

**Supplementary Information:**

The online version contains supplementary material available at 10.1007/s40820-026-02273-x.

## Graphical abstract

**Figure:**
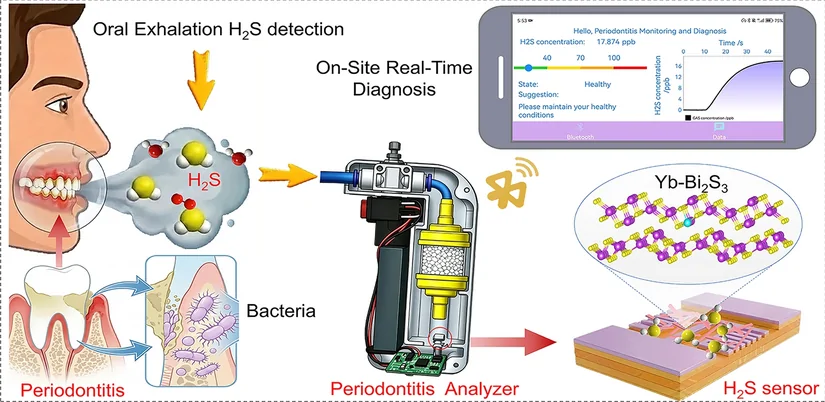

## Introduction

Periodontitis is a highly prevalent chronic inflammatory disease that damages periodontal supporting tissues, including the gums and alveolar bone [1, 2]. During disease progression, pathogenic oral bacteria metabolize sulfur‑containing amino acids and release H_2_S [3], which has been identified as a key biomarker closely associated with disease severity. In healthy individuals, oral exhalation H_2_S levels typically range from 8 to 40 ppb, whereas patients with periodontitis exhibit markedly elevated concentrations of 70–300 ppb due to intensified gingival inflammation and increased bacterial metabolic activity [4, 5]. Currently, the relationship between periodontitis-causing pathogens and released H_2_S is determined via gas chromatography [6–8], which suffers from drawbacks including large equipment requirements, complex operational procedures, and high testing costs. To reveal the intrinsic correlation, a portable H_2_S sensor would offer substantial convenience. Furthermore, the sensor holds significant clinical implications for periodontitis diagnosis [9–11]. It not only fulfills the need for immediate diagnosis in home and community settings but also enables non-invasive detection, thereby avoiding the discomfort associated with traditional methods for patients.

For the detection of trace H_2_S, metal oxide semiconductor (MOS) sensors have gained significant attention due to their low fabrication cost and well-established sensing mechanisms [12, 13]. However, traditional MOS sensors generally require high operating temperatures of 100–300 °C to achieve adequate response and recovery [14–16]. To mitigate this issue, auxiliary strategies such as -pulsed on-chip micro-heaters or UV/visible light activation have been employed to provide the necessary external energy [17]. Unfortunately, this approach not only significantly increases device power consumption and structural complexity but also poses safety hazards in oral detection scenarios [18]. An alternative approach involves the construction of multicomponent heterostructures, including MOS/MOS, MOS/MXene, and MOS/TMDC composites [19]. Nevertheless, their performance is strongly dependent on interface quality and reproducibility, while multi-step fabrication processes further add to the overall complexity [20, 21]. Accordingly, a single-phase material that enables efficient room-temperature H_2_S detection is urgently needed. The main challenges stem from insufficient gas adsorption and charge transfer under ambient conditions, together with the demands for stability and anti-interference ability in the complicated oral gas atmosphere [22, 23].

Bismuth sulfide (Bi_2_S_3_), a narrow-bandgap semiconductor (~ 1.24 eV), possesses high electrical conductivity and inherent H_2_S reactivity, which enables its distinct advantages in gas-sensing applications at room temperature [24, 25]. Metal–organic framework (MOF)-derived materials can further optimize the microstructure of Bi_2_S_3_. The porous framework and high surface area of MOF precursors can be well preserved after derivation, which accelerates gas diffusion and exposes abundant active sites [26, 27]. Nevertheless, pure MOF-derived Bi_2_S_3_ still exhibits weak reactivity and unsatisfactory sensitivity, limiting its practical application in accurate ppb-level H_2_S detection for periodontal diagnosis. Rare earth doping has been proven to be an effective strategy to address these limitations [28]. Currently, research on rare-earth-doped Bi_2_S_3_ is mainly concentrated on photoelectrochemistry, photocatalysis and photodetection, in which rare-earth 4*f* orbitals improve carrier separation. The potential of rare-earth doping to modulate surface properties and gas–solid interactions of Bi_2_S_3_ for H_2_S sensing remains unexplored. Among various rare-earth elements, ytterbium (Yb) was specifically chosen. Yb^3⁺^ possesses a smaller ionic radius (~ 0.868 Å) than Bi^3⁺^ (~ 1.03 Å), while other lanthanide ions (La^3⁺^, Ce^3⁺^, Nd^3⁺^) show radii close to Bi^3⁺^. The obvious ionic radius mismatch of Yb^3⁺^ induces severe lattice distortion and abundant surface defects [29]. Meanwhile, Yb^3⁺^ with unique 4*f* electronic configuration can effectively regulate the electronic structure of Bi_2_S_3_ and promote charge transfer-[30]. Furthermore, the incomplete 4*f* orbitals of Yb^3⁺^ strengthen the adsorption of oxygen and H_2_S molecules, thereby greatly improving the gas-sensing response.

Herein, we present an H_2_S sensor based on Yb-doped Bi_2_S_3_ (Yb-Bi_2_S_3_) nanoribbon derived from CAU-17, which enables on-site periodontitis diagnosis via detection of trace H_2_S gas in oral exhalation. The sensor exhibits segmented linear sensing across the 5 ppb–5 ppm concentration range, with a response of 3.31 to 100 ppb H_2_S and a response time of 11 s. It also demonstrates excellent resistance to interference from common oral gases. Density functional theory (DFT) calculations confirm that Yb-induced lattice distortion enhances both oxygen adsorption and oxygen anion generation efficiency while boosting selective H_2_S adsorption. Notably, Porphyromonas gingivalis was cultured and its H_2_S production levels were detected using this sensor, providing further evidence of the correlation between oral pathogens, periodontitis, and H_2_S levels. In clinical validation, the sensor successfully distinguished healthy individuals from periodontitis patients and provided diagnostic recommendations by assessing H_2_S concentrations in exhaled breath. This study provides a novel technical platform for rapid biomarker detection in periodontitis, offering significant reference for the development of intelligent medical diagnostic devices.

## Experimental Section

### Materials

Bismuth (III) nitrate pentahydrate (Bi(NO_3_)_3_.5H_2_O, ≥ 99.99%), trimesic acid (H_3_BTC, ≥ 99%), thiourea (CS(NH_2_)_2_, ≥ 99%), Ytterbium chloride hexahydrate (YbCl_3_·6H_2_O), N,N-dimethylformamide (DMF, ≥ 99.9%), methanol (MeOH, ≥ 99.9%) were purchased from Aladdin Biochemical Technology Co., Ltd. All reagents were of analytical grade and used without further purification.

### Synthesis of the CAU-17 and Yb-Doped Bi_2_S_3_ Hybrid

Synthesis of CAU-17: H_3_BTC (375 mg) was dissolved in a mixed solvent containing 24 mL DMF and 6 mL methanol, followed by the addition of 75 mg Bi(NO_3_)_3_·5H_2_O. After magnetic stirring for 1 h, the resulting mixture was transferred into a 50 mL Teflon-lined autoclave and heated at 120 °C for 24 h, yielding a white Bi‑MOF powder. The obtained product was washed sequentially with methanol, DMF, and ethanol, and then dried in a vacuum oven at 60 °C for 12 h.

The synthesized CAU-17 precursor was dispersed in 25 mL of DMF and stirred until fully homogenized. YbCl_3_·6H_2_O was then introduced at molar ratios of 0%, 0.2%, 0.5%, 1.0%, 1.5%, and 2.0%. Subsequently, 110 mg of thiourea was added to initiate the sulfidation process. After stirring for 1 h, the mixture was transferred to a 50 mL Teflon-lined autoclave and heated at 160 °C for 12 h. The final product was collected by centrifugation, thoroughly rinsed, and dried to obtain a black powder. The samples with different Yb doping levels were denoted as Bi_2_S_3_, Yb-Bi_2_S_3_-0.2%, Yb-Bi_2_S_3_-0.5%, Yb-Bi_2_S_3_-1.0%, Yb-Bi_2_S_3_-1.5%, and Yb-Bi_2_S_3_-2.0%, respectively. The sample labels correspond to the molar feed ratio of the YbCl_3_·6H_2_O precursor relative to Bi3⁺, and these are intended as nominal identifiers for the series of samples rather than absolute measures of actual doping concentration.

### Culture Method for Porphyromonas Gingivalis and Gas Collection

*Porphyromonas gingivalis* ATCC 33277 was cultured in Brain Heart Infusion (BHI) medium, wherein each 100 mL of medium contained 3.6 g BHI, 0.05 g yeast extract, 1.5 g agar, 500 *μ*L hemin, 100 *μ*L vitamin K, and 5 mL sheep blood. Inoculate the revived *p.g. *culture onto BHI solid medium and isolate using the streak method. Subculture every three days thereafter. An appropriate number of colonies were picked and inoculated into BHI liquid medium, followed by incubation in an anaerobic incubator at 37 °C for 48 h to prepare a bacterial suspension. Finally, the bacterial suspension was adjusted to a concentration of 1 × 10^8^ CFU mL^−1^ using phosphate-buffered saline (PBS) at pH 7.2 via the McFarland standard, and set aside for later use. The cultured *p.g.* suspension was aliquoted into 2 mL portions, sealed in 15 mL conical tubes, and further incubated for 12 h. Volatile gases above the bacterial suspension were extracted with a syringe and stored in 10 mL gas collection bags. The gas samples were analyzed by gas chromatography (GC) and Yb-Bi_2_S_3_ sensors, respectively.

### Characterization of Materials

The crystalline structures of the samples were analyzed by powder X-ray diffraction (XRD, Panalytical X’PERT) using Cu Kα radiation (*λ* = 0.15418 nm) over a 2θ range of 10°–90°. Morphological characteristics and elemental distributions were examined via scanning electron microscopy (SEM, VEGA3 LMH) and high-resolution transmission electron microscopy (HRTEM, Tecnai G2 F30, USA). The chemical compositions and valence states of the elements were investigated by X-ray photoelectron spectroscopy (XPS) using a Thermo Scientific K-Alpha spectrometer. Raman spectra were collected with a Renishaw inVia spectrometer equipped with a 532 nm excitation laser at an incident power of 5 mW. Specific surface areas were determined through Brunauer–Emmett–Teller (BET) analysis using a Micromeritics ASAP 2020 instrument. Electron paramagnetic resonance (EPR) spectra were recorded on a JEOL X-band spectrometer (JES-FA200). The work functions of Bi_2_S_3_ and Yb-Bi_2_S_3_ samples were measured using ultraviolet photoelectron spectroscopy (UPS) on a Thermo Scientific NEXSA system and Kelvin probe force microscopy (KPFM) on an Oxford Instruments Asylum Research MFP-3D Orange atomic force microscope. Fourier-transform infrared spectroscopy (FTIR) measurements were performed on a Shimadzu FTIR spectrophotometer (NEXUS 370). Gas chromatography testing employs an Agilent 8890 gas chromatograph equipped with a sulfur chemiluminescence SCD detector.

### Gas Sensor Fabrication and Measurements

The gas sensor was fabricated using a drop-coating method with the synthesized sensing materials and an interdigitated electrode. The interdigitated electrodes were fabricated on a 500 *μ*m thick aluminum oxide (Al_2_O_3_) ceramic substrate, with overall dimensions of 7 mm × 4 mm × 0.5 mm. The line width and spacing of the interdigitated electrodes were both 60 *μ*m, and the effective sensing area was 4.0 mm × 4.0 mm. The copper electrode layer has a thickness of 18 *μ*m, onto which a 4-*μ*m-thick electroless nickel layer and a 0.05-*μ*m-thick electroless gold layer were sequentially deposited to ensure reliable electrical contact and chemical stability. For the sensor fabrication process, 20 mg of powder sample was dispersed in 1 mL of ethanol and ultrasonicated for 30 min to obtain a uniform suspension. Then, 20 *μ*L of the suspension was pipetted and drop-cast onto the interdigitated electrode. To enhance the interfacial contact between the sensing material and the electrode, the coated substrate was dried in a vacuum oven at 60 °C for 2 h. Before performing gas-sensing measurements, the device was aged at room temperature under a 3 V bias for 10 h to ensure operational stability.

Gas-sensing performance was evaluated using a custom-built gas analysis system (Fig. S1). Real-time conductivity changes were monitored and recorded using a Source Meter (Keithley 2450). Ambient air served as the baseline gas, and defined volumes of H_2_S were injected into a 4 L chamber using a micro-syringe to achieve the desired target gas concentrations. Unless otherwise specified, all measurements were conducted at room temperature with a natural relative humidity (RH) of approximately 20%–30%. The sensor’s response toward H_2_S was also systematically examined under varying humidity conditions. Different humidity environments were established by placing saturated salt solutions in the sealed chamber and allowing sufficient equilibration time. Specifically, saturated solutions of LiCl, MgCl_2_, K_2_CO_3_, CuCl_2_, and KNO_3_ were used to generate different RH levels, and the actual humidity inside the chamber was calibrated with a standard humidity sensor (Vaisala GMP110). The sensing response (*S*) was calculated as *S* = *R*_a_/*R*_g_ for reducing gases and *S* = *R*_g_/*R*_a_ for oxidizing gases, where *R*_a_ and *R*_g_ denote the sensor resistance in air and in the target gas atmosphere, respectively. Given that H_2_S is a reducing gas, all response values reported in this study were calculated strictly according to the formula *S* = *R*_a_/*R*_g._ The response time and recovery time were defined as the time required to reach 90% of the total resistance change during the adsorption and desorption processes.

### DFT Calculations

The geometric and electronic structures were investigated via DFT calculations using the Vienna Ab initio Simulation Package (VASP) with the PBE exchange–correlation functional. A DFT-D3-BJ dispersion correction was applied to account for van der Waals interactions. Geometry optimization was carried out using a slab model in which three atomic layers were allowed to relax. The slab model was constructed based on the (130) crystal plane of orthorhombic Bi_2_S_3_, which was identified by high-resolution TEM as the dominant exposed facet in the synthesized nanoribbons. To ensure thermodynamic stability under experimental conditions, the surface was terminated with sulfur atoms rather than bismuth atoms. The plane-wave cutoff energy was set to 400 eV. A 2 × 2 × 1 Monkhorst–Pack k-point grid was used for Brillouin-zone sampling. The energy and force convergence thresholds were set to 1 × 10⁻^5^ eV and 0.02 eV Å⁻^1^, respectively. Spin-polarized calculations were performed throughout this work. VASPKIT was employed for post-processing of the computational data.

### Patient Breath Testing Experiments

A portable breath analysis device was developed based on the Yb-Bi_2_S_3_-1.5% sample. To evaluate the practical applicability of the device, the breath responses of patients and healthy individuals were tested. This study was approved by the Institutional Review Board (IRB) of The Second Affiliated Hospital of Harbin Medical University (Approval No. KY2025-237). All participants provided written informed consent prior to participation. The study was conducted in accordance with the Declaration of Helsinki.

1. Subject Recruitment, Inclusion, and Exclusion Criteria
  Inclusion criteria: For the periodontitis group, patients were diagnosed with moderate to severe chronic periodontitis, defined as the presence of interproximal clinical attachment loss ≥ 3 mm and probing pocket depths ≥ 5 mm at multiple sites. For the healthy control group, subjects showed no clinical signs of periodontal disease (probing depth ≤ 3 mm, no bleeding on probing, no alveolar bone resorption), had no other oral diseases such as dental caries, oral ulcers, or tonsillitis, and had no systemic diseases that could affect oral health or the composition of exhaled breath.
  Exclusion criteria: To minimize confounding sources of exhaled H_2_S, subjects with the following conditions were strictly excluded: (i) Participants with a history of gastrointestinal diseases (e.g., gastritis, peptic ulcers, or Helicobacter pylori infection) or respiratory diseases (e.g., asthma or chronic obstructive pulmonary disease); (ii) those who had used antibiotics, anti-inflammatory drugs, or oral antiseptics within 7 days prior to testing; (iii) current smokers or alcohol consumers; (iv) pregnant or breastfeeding women, as well as participants unable to cooperate with the breath sampling procedure.
2. Oral Environment Control Prior to Sampling
  All participants were required to strictly follow the preparation schedule to minimize intra- and inter-individual variability. Participants were instructed to: avoid consuming spicy or irritating foods as well as dairy products for 24 h prior to testing; on the morning of the test, limit brushing, flossing, or using mouthwash to ensure a washout period of at least 8 h since the last oral hygiene routine; strictly fast for at least 2 h (except for plain water), and avoid eating, chewing gum, and drinking any flavored beverages.
3. Standardized Sampling Procedure
  No mouth rinsing was performed prior to sampling to avoid artificially lowering endogenous H_2_S levels in the oral cavity. Before exhaling, subjects sat quietly with their mouths closed for three minutes to reach a steady state, allowing the gas in the oral cavity to accumulate to a representative steady-state concentration. During sampling, each subject first took a deep nasal breath and held it for 5 s to ensure that the gases in the oral cavity and upper respiratory tract had fully reached equilibrium. Subsequently, the subject exhaled slowly and continuously through the sampling port at a constant flow rate of 3–5 L min^−1^.

## Results and Discussion

### Monitoring and Sensing of Periodontitis Associated with H_2_S

**Fig. 1:**
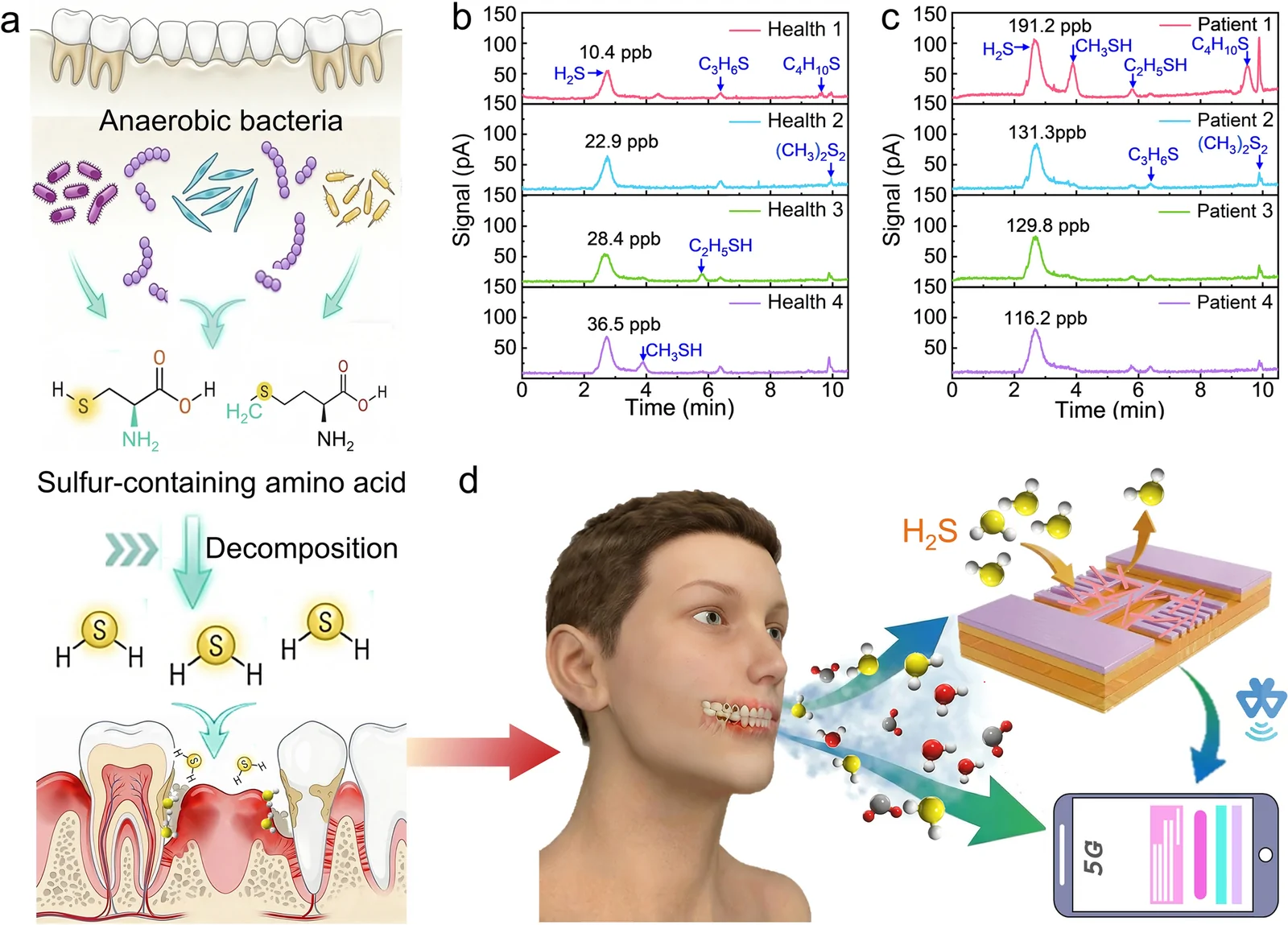
Conception of on-site periodontitis diagnosis via detection of trace H_2_S in oral exhalation. **a** Schematic diagram of H_2_S production by oral pathogenic bacteria in periodontitis patients. **b** Gas chromatography (GC) analysis of sulfur-containing gases in oral exhalation from four healthy subjects (Health 1–4). **c** GC analysis of sulfur-containing gases in oral exhalation from four periodontitis patients (Patient 1–4). **d** Schematic of H_2_S sensor for diagnosing periodontitis through oral exhaled gas

The oral exhaled H_2_S level in periodontitis patients is significantly higher than that in healthy individuals, supporting H_2_S as a potential biomarker for periodontitis progression [4]. Figure 1 systematically illustrates the metabolic sources of periodontitis-associated H_2_S, its clinical exhalation characteristics, and non-invasive monitoring via gas sensors. The core pathways of oral H_2_S production in periodontitis patients (Fig. 1a) are clarified. Within the anaerobic microenvironment of periodontal pockets, anaerobic bacteria colonizing tooth surfaces degrade sulfur-containing amino acids (e.g., cysteine, methionine) derived from saliva, gingival crevicular fluid, and epithelial cells [31, 32]. Continuous degradation of these amino acids generates local H_2_S, which contributes to periodontal tissue inflammatory damage [33]. This metabolic process provides a molecular basis for local H_2_S accumulation in the periodontal microenvironment and explains the metabolic origin of toxic gases in inflamed regions.

To verify the difference in oral H_2_S exhalation between periodontitis patients and healthy individuals, the sulfur-containing gases in exhaled breath were analyzed from 4 healthy subjects (Health 1–4) and 4 periodontitis patients (Patient 1–4) using gas chromatography (GC) (Fig. 1b, c). The oral exhaled sulfur-containing gases mainly include H_2_S and trace amounts of methyl mercaptan (CH_3_SH), ethyl mercaptan (C_2_H_5_SH), dimethyl sulfide (C_2_H_6_S), ethanethiol (C_4_H_1_₀S), and dimethyl disulfide ((CH_3_)_2_S_2_) (Fig. 1b, c). For the healthy subjects, the H_2_S concentrations ranged from 10.4 to 36.5 ppb (Fig. 1b), whereas periodontitis patients had significantly elevated levels of 116.2–191.2 ppb (Fig. 1c). The significant differences in exhaled H_2_S were observed between the two groups, confirming a positive correlation between H_2_S concentration and periodontitis severity. These results further validate H_2_S as a potential biomarker for periodontitis progression.

According to the experimental results, we present a real-time exhalation monitor equipped with an H_2_S sensor for point-of-care monitoring of periodontitis patients (Fig. 1d). The sensor detects exhaled H_2_S on-site, and the response signal is transmitted wirelessly (e.g., via 5G) to a mobile terminal, where concentration characteristic curves are displayed in real time. The system enables non-invasive, real-time monitoring and auxiliary diagnosis of periodontitis. The proposed approach not only offers a practical tool for bedside monitoring and long-term follow-up of periodontitis but also extends the clinical applicability of non-invasive diagnostic technologies in oral healthcare.

### Characterization of Sensitive Materials for H_2_S Sensors

**Fig. 2:**
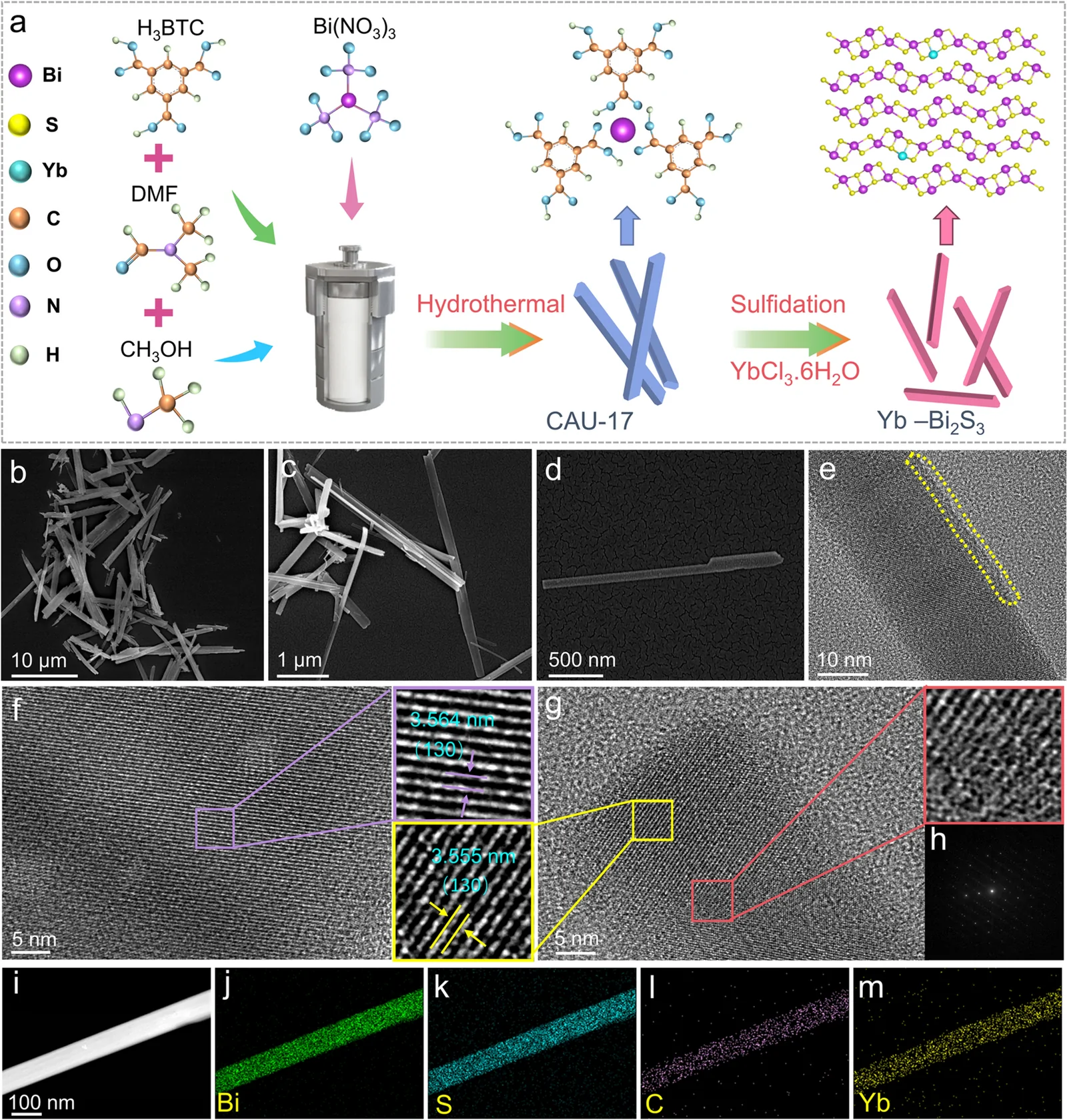
Synthesis routes and characterization of Bi_2_S_3_ and Yb-Bi_2_S_3_ samples. **a** Schematic diagram of the synthesis route for Yb-Bi_2_S_3_. **b-d** SEM images of CAU-17, Bi_2_S_3_, and Yb-Bi_2_S_3_-1.5%, respectively. **e** TEM image of the Yb-Bi_2_S_3_-1.5% sample. **f, g** High-resolution TEM image of Bi_2_S_3_ and Yb-Bi_2_S_3_-1.5% (Inset: corresponding crystal planes and lattice spacings). **h** SAED pattern of Yb-Bi_2_S_3_-1.5%. **i-m** Elemental mapping of Yb-Bi_2_S_3_-1.5% with Bi, S, C, Yb elements distribution

For trace H_2_S sensing, a highly efficient sensing material was developed in the experiments. As illustrated in Fig. 2a, Bi_2_S_3_ and Yb-Bi_2_S_3_ samples were synthesized through polymerization followed by a sulfurization process. In brief, a Bi-based metal–organic framework (CAU-17) was synthesized as the precursor via simple coordination polymerization using H_3_BTC as the organic ligand. The obtained CAU-17 displayed a well-defined fibrous ribbon-like morphology with lengths of approximately 20 *μ*m (Fig. 2b). Subsequently, thiourea was employed as the sulfur source to induce in situ sulfurization of the Bi^3⁺^ centers within CAU-17, resulting in the formation of Bi_2_S_3_ (Fig. 2c). Compared with CAU-17, the resulting Bi_2_S_3_ exhibited shortened nanoribbon structures with lengths of about 4 *μ*m and widths of around 40 nm. In contrast to Bi_2_S_3_, Yb-doped Bi_2_S_3_ samples preserved the overall morphology and particle dimensions (Figs. 2d and S2), indicating that Yb incorporation did not induce significant structural alterations. The TEM image in Fig. S3 further verifies the nanoribbon morphology of Yb-Bi_2_S_3_, consistent with the SEM observations. Notably, the high-resolution TEM image (Fig. 2e) reveals the presence of a coating nanolayer derived from organic ligands, which clearly wraps the Bi_2_S_3_ nanoribbon. This novel composite structure of nanocrystals and organic carbon layers can effectively enhance electron mobility and material stability [30]. For the pure Bi_2_S_3_ sample, the high-resolution TEM image reveals clear lattice fringes with an lattice spacing of 3.564 Å, corresponding to the (130) crystal plane of Bi_2_S_3_ (purple box in Fig. 2f). In contrast, the Yb-doped Bi_2_S_3_ sample exhibits a slightly reduced lattice spacing of 3.555 Å for the same (130) plane (yellow box in Fig. 2g). This contraction is attributed to the replacement of Bi^3⁺^ with larger ionic radius (~ 1.03 Å) by Yb^3⁺^ ions with smaller ionic radius (~ 0.868 Å) [34]. Moreover, the distorted lattice fringes highlighted in the pink box provide direct evidence of lattice distortion within Yb-Bi_2_S_3_ nanocrystals, further confirming the successful incorporation of Yb dopants [35]. The SAED pattern shown in Fig. 2h displays well-defined orthorhombic diffraction spots, indicative of the single-crystalline nature of the material. Elemental mapping images (Fig. 2i–m) reveal that Bi, S, Yb, and C are uniformly distributed across the whole nanoribbon. Quantitative EDS analysis shows that the actual Yb atomic loading is 1.47 at%, which is highly consistent with the nominal loading ratio, indicating a high doping efficiency. The uniform carbon distribution (Fig. 2l) further confirms the presence of an organic nanolayer coating within the MOF-derived Bi_2_S_3_. These results conclusively demonstrate the successful doping of Yb into the Bi_2_S_3_ lattice and the accompanying lattice distortion induced by Yb incorporation.

**Fig. 3:**
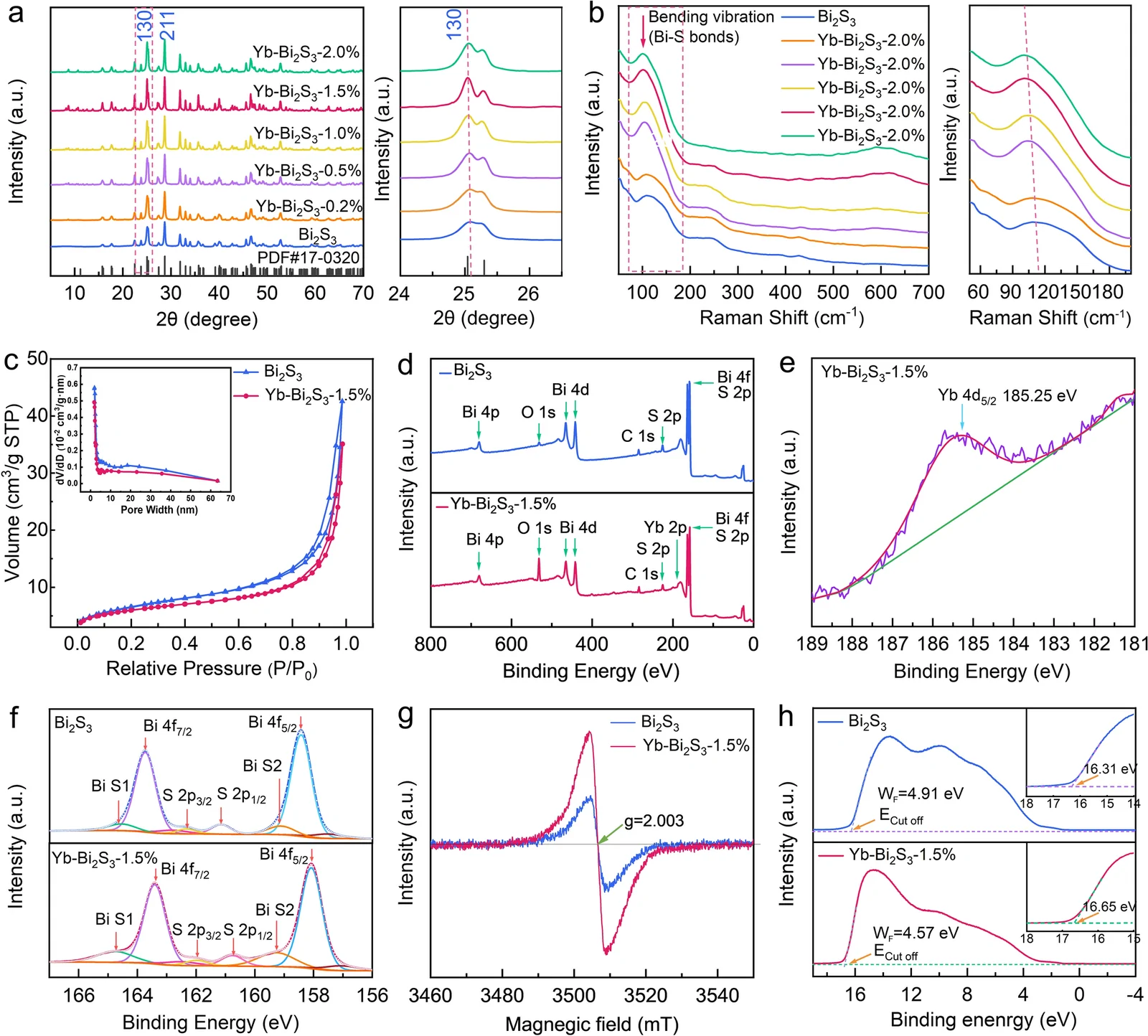
Characterization of crystal structure and energy band of Bi_2_S_3_ and Yb-Bi_2_S_3_. **a** XRD spectra and **b** Raman spectra of Bi_2_S_3_ and Yb-Bi_2_S_3_ samples with various doping concentrations. **c** N_2_ adsorption–desorption isotherms of Bi_2_S_3_ and Yb-Bi_2_S_3_-1.5% samples. The inset shows the pore size distributions of the two samples. **d** XPS survey spectra of Bi_2_S_3_ and Yb-Bi_2_S_3_-1.5% samples. **e** High-resolution XPS spectra of Yb 4*d* of Yb-Bi_2_S_3_-1.5% sample.** f** High-resolution XPS spectra of Bi 4f and S 2*p* of Bi_2_S_3_ and Yb-Bi_2_S_3_-1.5% samples.** g** EPR spectra and** h** UPS spectra of Bi_2_S_3_ and Yb-Bi_2_S_3_-1.5% samples

Figure 3a shows the XRD patterns of Bi_2_S_3_ and Yb-Bi_2_S_3_ samples with different Yb doping levels. All samples exhibit characteristic diffraction peaks of orthorhombic Bi_2_S_3_, with the (130) and (211) planes being dominant. As the Yb content increases, the diffraction peaks of Yb-Bi_2_S_3_ become stronger and narrower, indicating improved crystallinity and structural stability. However, at 2% Yb doping, excessive lattice distortion causes the diffraction peak shape to broaden slightly [36]. The peak shift toward lower diffraction angles is attributed to lattice contraction caused by the substitution of larger Bi^3⁺^ with smaller Yb_3⁺_. Raman spectra (Fig. 3b) collected in the 100–700 cm^−1^ range further confirm the structural modification induced by Yb doping. The pure Bi_2_S_3_ shows a broad and weak Raman characteristic peak at 113 cm^−1^ associated with the Bi–S bond bending vibration and interlayer shear mode in orthorhombic Bi_2_S_3_. With increasing Yb content, the Raman peak becomes sharper and more intense, indicating that the defect levels introduced by doping enhance electron–phonon coupling while improving the material’s crystallinity [30]. The peak shift (from 113 to 102 cm^−1^), consistent with the XRD results, also arises from lattice distortion induced by Yb3⁺ incorporation. The pore structures and specific surface areas of pure Bi_2_S_3_ and Yb-Bi_2_S_3_ were analyzed using nitrogen adsorption–desorption isotherms (Fig. 3c). Both samples exhibit typical Type IV isotherms with pronounced hysteresis loops, indicating the mesoporous feature of the materials. The pure Bi_2_S_3_ sample shows a concentrated distribution of small mesopores (average pore size of 11.57 nm) with a specific surface area of 23.8 m^2^ g^−1^, which is significantly higher than previously reported Bi_2_S_3_ materials [37, 38]. This indicates that the synthesized Bi_2_S_3_ inherits the morphological characteristics of its precursor CAU-17, forming a rich pore/void structure that provides more gas adsorption sites. Meanwhile, the Yb-Bi_2_S_3_-1.5% sample shows a slightly lower surface area of 20.5 m^2^ g^−1^ and larger average pore sizes (12.63 nm). The slight reduction is likely due to Yb3⁺ serving as heterogeneous nucleation sites, promoting the growth of larger aggregate particles.

Figure 3d presents the XPS survey spectra of pure Bi_2_S_3_ and the Yb-Bi_2_S_3_-1.5% samples. In pure Bi_2_S_3_, the characteristic peaks of Bi 4*p*, Bi 4*d*, O 1*s*, C 1*s*, and S 2*p* are observed, consistent with its intrinsic elemental composition [32]. In contrast, the Yb-Bi_2_S_3_-1.5% displays a distinct Yb 4*d* signal at approximately 185 eV, confirming the successful incorporation of Yb into Bi_2_S_3_. The sample yields a surface Yb concentration of approximately 0.78 at%. The lower surface concentration measured by XPS can be attributed to the shallow sampling depth and the tendency of Yb^3+^ to occupy bulk rather than surface lattice sites.The high-resolution Yb 4*d* spectrum (Fig. 3e) shows a Yb 4*d*_5_/_2_ peak at 185.26 eV, which corresponds to the binding energy of Yb3⁺, further verifying the oxidation state of the doped Yb [39]. Figure 3f compares the high-resolution XPS spectra of Bi 4*f* and S 2*p* for both samples. In pure Bi_2_S_3_, the Bi 4*f*_7_/_2_ and Bi 4*f*_5/2_ peaks are located at 163.74 and 158.43 eV, while the S 2*p*_3/2_ and S 2*p*_1/2_ peaks at 162.33 and 161.14 eV are characteristic of lattice S_2⁻_ [39, 40]. Weak shoulder peaks (Bi S1 and Bi S2) are attributed to unsaturated Bi sites associated with surface sulfur vacancies [41]. After Yb doping, the Bi 4*f* peaks shift to higher binding energies, whereas the S 2*p* peaks shift to lower energies, accompanied by a significant increase in the intensity of the shoulder peaks. Yb^3⁺^ substitution for Bi^3⁺^ shortens the Bi-S bond length and increases its bond energy, reducing the electron density of the Bi atom (increasing its binding energy) while increasing that of the S atom. Concurrently, Yb doping may induce sulfur vacancy defects, leading to an elevated number of unsaturated coordination Bi sites on the surface and enhanced shoulder peak contributions. The observation can be further confirmed by EPR spectra (Fig. 3g), where the characteristic signal (g ≈ 2.003) is attributed to electrons trapped in sulfur vacancies [42]. The significantly higher intensity in Yb-Bi_2_S_3_ confirms that Yb^3⁺^ substitution promotes sulfur vacancies formation via lattice distortion and bond destabilization. These abundant vacancies act as critical active sites for the adsorption and activation of O_2_ and H_2_S, serving as the structural basis for the enhanced gas-sensing performance. In addition, a comparative XPS C 1*s* analysis further confirms the MOF origin of the carbon layer. Unlike a control Yb-doped Bi_2_S_3_ sample prepared without a MOF precursor, the CAU-17-derived Yb-Bi_2_S_3_-1.5% displays an additional prominent O–C=O component at ~ 288.5 eV (Fig. S4). This carboxylate signature derives from residual H_3_BTC ligand. Consistent with the literature on MOF-derived sensing materials, this carbon coating forms a tight interface with Bi_2_S_3_ and may enhance the mechanical and chemical stability of the sensing material [43, 44].

To further explore the energy band structures of these samples, UPS, KPFM, and UV–vis DRS were employed, and the results are accumulated in Figs. 3h, S5, and S6. The secondary electron cutoff energy (E_cut-off_) was determined using the high binding energy region of the UPS spectrum, revealing a work function of 4.91 eV for the pristine Bi_2_S_3_ material, while that of Yb-Bi_2_S_3_-1.5% decreased to 4.57 eV (Fig. 3h) (work function *φ* = 21.22 eV-E_cut-off_, where 21.22 eV is the energy of the UV-source). The KPFM surface potential maps (Fig. S5) reveal a uniformly lower surface potential for Yb-Bi_2_S_3_‑1.5% relative to pristine Bi_2_S_3_, corresponding to an estimated work function reduction of approximately 0.25 eV, in good agreement with the UPS-derived difference. In addition, the optical bandgaps of Bi_2_S_3_ and Yb-Bi_2_S_3_-1.5% were calculated to be 1.35 and 1.12 eV, respectively, using the Kubelka–Munk equation (Fig. S6). Obviously, the Yb-Bi_2_S_3_-1.5% sample exhibits a reduced work function, a reduced bandgap, and enhanced conductivity compared to pristine Bi_2_S_3_. Notably, for the n-type semiconductor Bi_2_S_3_, the chemical adsorption of O_2_ on the surface essentially involves the extraction of electrons from the material’s conduction band. Yb doping reduces the work function of Bi_2_S_3_, which means that the Fermi level at the material’s surface is raised. This results in conduction band electrons having higher energy and a stronger tendency to escape, thereby significantly enhancing the thermodynamic driving force for electron transfer to adsorbed O_2_ molecules. Furthermore, the lower work function allows for a higher equilibrium concentration of surface O_2_⁻ to exist stably per unit area of the material, indicating that the sensing material accumulates a greater reserve of reactive oxygen species. Upon contact with H_2_S gas, this enables more thorough redox reactions, resulting in larger resistance modulation amplitudes and higher response values [45–47].

### Sensing Performance of the Yb-Bi_2_S_3_ Sensor

**Fig. 4:**
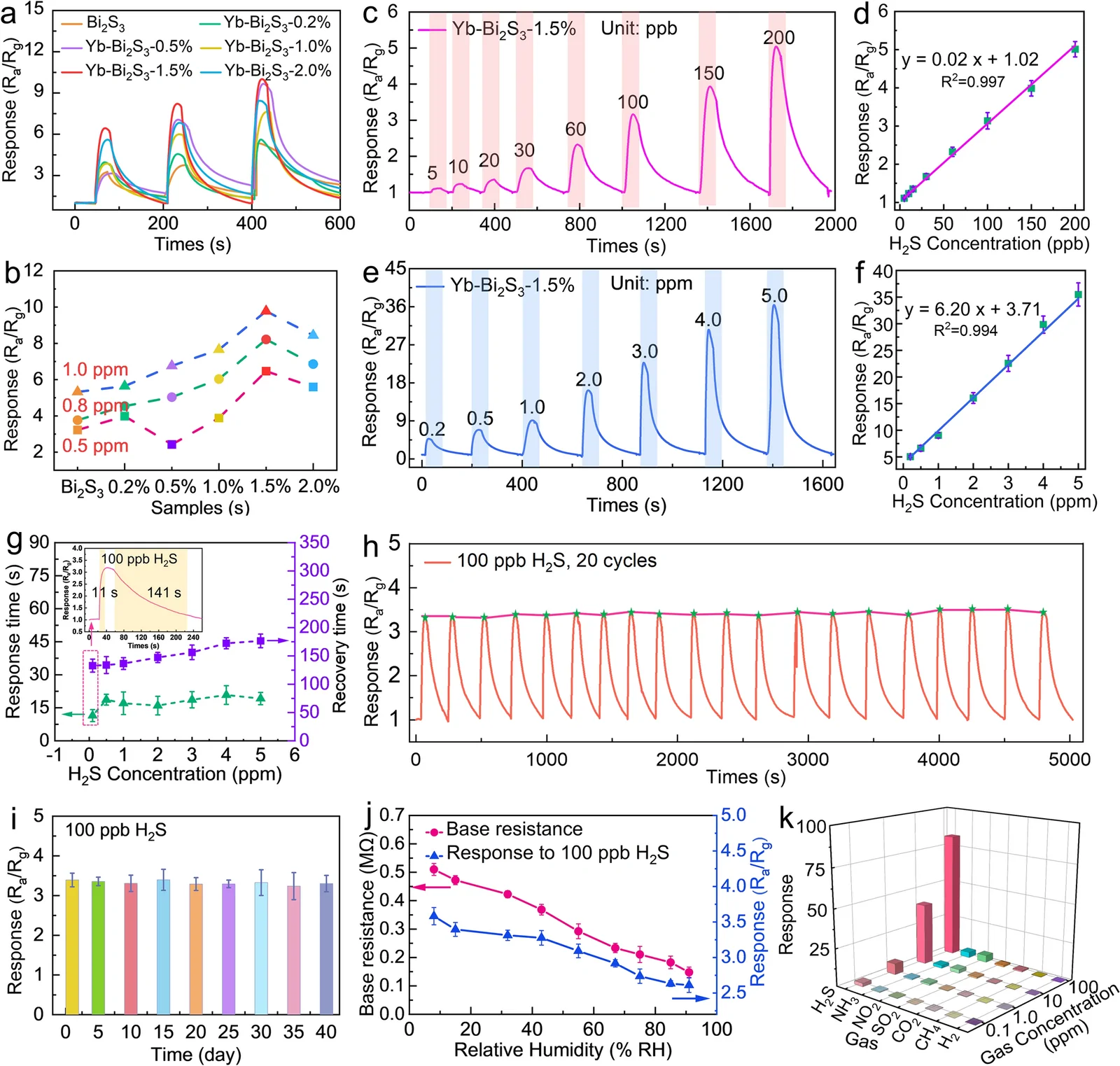
Sensing performance of Bi_2_S_3_ and Yb-Bi_2_S_3_ in air at room temperature (all error bars presented in this figure represent standard deviation). **a** Dynamic response curves toward H_2_S and **b** response statistics of Bi_2_S_3_ and Yb-doped Bi_2_S_3_ samples at different Yb doping concentrations. **c** Dynamic response-recovery curves of Yb-Bi_2_S_3_-1.5% in the range of 5–200 ppb. **d** Response with respect to the H_2_S concentration in the range of 5–200 ppb. **e** Dynamic response-recovery curves of Yb-Bi_2_S_3_-1.5% in the range of 0.2–5 ppm.** f** Response with respect to the H_2_S concentration in the range of 0.2–5 ppm. **g** Response (left y-axis) and recovery (right y-axis) times of Yb-Bi_2_S_3_-1.5% sensor toward different H_2_S concentrations, with the inset presenting its dynamic response–recovery curve at 100 ppb. **h** Repeatability test of Yb-Bi_2_S_3_-1.5% toward 100 ppb H_2_S for 20 cycles.** i** Long-term stability to 100 ppb H_2_S within 40 days. **j** Base resistance and response to 100 ppb H_2_S of Yb-Bi_2_S_3_-1.5% as a function of relative humidity. **k** Selective response toward NO_2_, SO_2_, CO_2_, CO, NH_3_, H_2_ and H_2_S at four distinct concentration levels: 0.1, 1.0, 10, and 100 ppm

The gas-sensing performances of the samples were evaluated in air at room temperature environment (25 °C, 20%–30% RH). Herein, the response to H_2_S is defined as* R*ₐ/*R*_g_, where *R*ₐ and *R*_g_ are the sensor resistances measured in air and in the target gas, respectively. Figure 4a, b show the sensing response characteristics of Bi_2_S_3_ and Yb-doped Bi_2_S_3_ samples with different Yb concentrations under 0.5, 0.8, and 1.0 ppm H_2_S atmospheres. The pure Bi_2_S_3_ exhibited the lowest response levels at all three concentrations, indicating that Yb doping significantly enhances sensitivity. As the Yb doping concentration increased to Yb‑Bi_2_S_3_‑1.5%, the response at each concentration showed an upward trend. Further increasing the doping level (Yb‑Bi_2_S_3_‑2.0%) led to a decline in response (Fig. 4a, b). This trend may stem from the fact that moderate Yb doping induces suitable lattice distortion, which optimizes the electronic structure and promotes both H_2_S adsorption and charge transfer. In contrast, excessive doping triggers grain agglomeration and a reduced specific surface area. Additionally, the increased defects exacerbate carrier scattering, diminishing signal transmission efficiency during H_2_S adsorption–desorption processes. Therefore, Yb-Bi_2_S_3_-1.5% emerges as the optimal doping concentration sample and is suitable for subsequent in-depth testing of H_2_S gas-sensing responsiveness.

Figure 4c–f displays the dynamic response curves of Yb-Bi_2_S_3_-1.5% toward H_2_S over two concentration ranges along with their corresponding linear fits. In the 5–200 ppb range (Fig. 4c), the response increases gradually with rising H_2_S concentration. It exhibits rapid response-recovery behavior and excellent reversibility at all levels, demonstrating high sensitivity toward trace H_2_S exhalation in the oral cavity of both healthy individuals and patients. The linear fitting curve in Fig. 4d indicates a strong linear correlation between response and concentration, with a sensitivity of 0.02/ppb (i.e., 20/ppm). Furthermore, the sensor consistently responds to trace H_2_S at 5 ppb across multiple measurements (Fig. S7), confirming its detection limit below 5 ppb and further validating its reliable quantitative detection capability for exhaled H_2_S at the ppb level. As the H_2_S concentration increases from 0.2 to 5.0 ppm, the response rises from 4.86 to 36.50 while maintaining rapid and reversible response–recovery behavior, indicating stable sensing performance over a wide concentration range (Fig. 4e). The linear fitting curve in Fig. 4f further demonstrates excellent linearity in this region, with a sensitivity of 6.20/ppm. The breakpoint near 200 ppb was empirically determined from multiple replicate response–concentration measurements, which consistently revealed a clear slope change in the response curve at this concentration. The higher apparent sensitivity in the low-concentration region is likely due to trace H_2_S molecules preferentially occupying the most active surface sites (such as sulfur vacancies), leading to more efficient charge transfer and resulting in stronger resistance modulation. As directly compared in Fig. S8, segmented fitting (*R*^2^ = 0.997, 0.994) yields a superior fit and accuracy over single-range fitting (*R*^2 ^= 0.949). Since oral H_2_S levels for periodontitis diagnosis typically fall in the low-concentration range, segmented fitting better describes the sensing behavior under clinically relevant conditions.

In practical applications of periodontitis detection, the response time, repeatability, stability, and selectivity of H_2_S sensors are also critical. Considering the H_2_S levels in the oral cavities of both healthy individuals and patients, a concentration of 100 ppb H_2_S was selected to validate the aforementioned sensor metrics. Figure 4g displays the response time (left y-axis) and recovery time (right y-axis) of Yb-Bi_2_S_3_-1.5% toward different H_2_S concentrations, with the inset presenting its dynamic response–recovery curve at 100 ppb. Across the 0–5 ppm range, the response time remains low (about 15 s or less), indicating fast reaction kinetics. The recovery time increases gradually with higher H_2_S concentrations but stays within 150 s, likely due to enhanced adsorption of H_2_S on active sites at elevated concentrations. Overall, Yb-Bi_2_S_3_-1.5% demonstrates fast response and stable recovery behavior, meeting the requirements of practical H_2_S sensing applications. In addition, the stability of the sensor was evaluated through testing over 20 consecutive cycles under 100 ppb H_2_S (Fig. 4h). The sensor maintains stable response values throughout all cycles with peak responses consistently ranging from 3.29 to 3.53, showing no noticeable attenuation or fluctuation. This confirms the excellent repeatability of the device. The long-term stability of Yb-Bi_2_S_3_-1.5% was further monitored by measuring its response to 100 ppb H_2_S over 40 days (Fig. 4i). The sensor devices were stored in a sealed desiccator under ambient room temperature (~ 25 °C) without any specific humidity control(~ 20–30% RH). From Day 1 to Day 40, the sensor continuously exhibited high response values above 3, with no observable decline and small error bars across all measurements, indicating that the Yb-Bi_2_S_3_-1.5% lattice remains stable without grain coarsening or structural degradation during storage. It can meet the performance requirements for long-term sensor operation in actual periodontitis detection scenarios. The influence of humidity on the sensor was evaluated by measuring the baseline resistance and the response to 100 ppb H_2_S at different relative humidity levels (Fig. 4j). As humidity increased from 8% to 91% RH, the baseline resistance decreased from 510 to 148 kΩ, accompanied by a gradual reduction in H_2_S response. Under high humidity, adsorbed water adsorption competes with H_2_S for active sites on the Yb-Bi_2_S_3_ surface, weakening H_2_S adsorption and reducing sensor responsivity. Nevertheless, even at 91% RH, the sensor retains a response > 2.5, demonstrating reliable detection under humid conditions. For practical detection of periodontitis, a drying tube can be installed at the front end of the detector to compensate for humidity effects.

The 3D histogram (Fig. 4k) demonstrates the response of Yb-Bi_2_S_3_-1.5% to H_2_S and various interfering gases (NH_3_, NO_2_, SO_2_, CO_2_, CH_4_, H_2_, etc.). Under identical testing conditions, it exhibits significantly higher response specificity toward H_2_S compared to interfering gases. Furthermore, additional selectivity tests were conducted on typical VOCs in oral breath (including ethanol, acetone, and formaldehyde) as well as VSCs clinically associated with periodontitis (including CH_3_SH, C_2_H_5_SH, and (CH_3_)_2_S) (Fig. S9). The results clearly demonstrate that the sensor responds much more strongly to H_2_S, with a selectivity toward VOCs > 10 (R_H2S_/ R_VOCs_ > 10) and a selectivity toward VSCs > 4 (R_H2S_/ R_VSCs_ > 4). In summary, the Yb-Bi_2_S_3_-1.5% sensor effectively suppresses interference from other oral gases, making it suitable for the specific detection of H_2_S in the exhaled breath of periodontitis patients. In the hysteresis test (Fig. S10), the device demonstrated excellent consistency and reversibility of response signals during cycles of gradually increasing and decreasing concentrations. The sensor’s low hysteresis characteristic effectively minimizes response deviations during multiple H_2_S measurements for periodontitis. To simulate actual clinical use, the Yb-Bi_2_S_3_-1.5% sensor underwent 30 consecutive exhalation sampling cycles over a 5-day period (6 cycles per day). The sensor maintained stable response values over 5 days, with average response values of 3.20 ± 0.09, 3.17 ± 0.06, 3.19 ± 0.11, 3.12 ± 0.11, and 2.99 ± 0.12, respectively (Fig. S11). It showed no systematic drift or performance degradation, indicating that the sensor possesses excellent operational stability under repeated simulated exhalation exposure conditions. To evaluate the reproducibility of sensor fabrication, five devices were fabricated from the same batch of Yb-Bi_2_S_3_-1.5% material and tested with 100 ppb H_2_S (Fig. S12). The relative standard deviations (RSD) for the sensor response and baseline resistance within the same batch were 6.60% and 8.53%, respectively. Furthermore, multiple sensors fabricated from three different batches exhibited average resistances and average response values with inter-batch RSDs of 10.69% and 6.24%, respectively, confirming the reliability of the material synthesis and device fabrication processes. Based on the aforementioned gas-sensing characteristics, the Yb-Bi_2_S_3_-1.5% sensor demonstrates the capability to detect trace amounts of H_2_S in exhaled breath with outstanding stability and selectivity. For comprehensive performance evaluation, a comparison with recently reported H_2_S sensors based on various sensitive materials is presented in Table S1. The summarized key parameters demonstrate that the Yb-doped Bi_2_S_3_ sensor exhibits outstanding room-temperature sensing performance, further highlighting its practical potential for future applications in periodontitis detection.

### Gas-Sensing Mechanism of the Yb-Bi_2_S_3_ Sensor

**Fig. 5:**
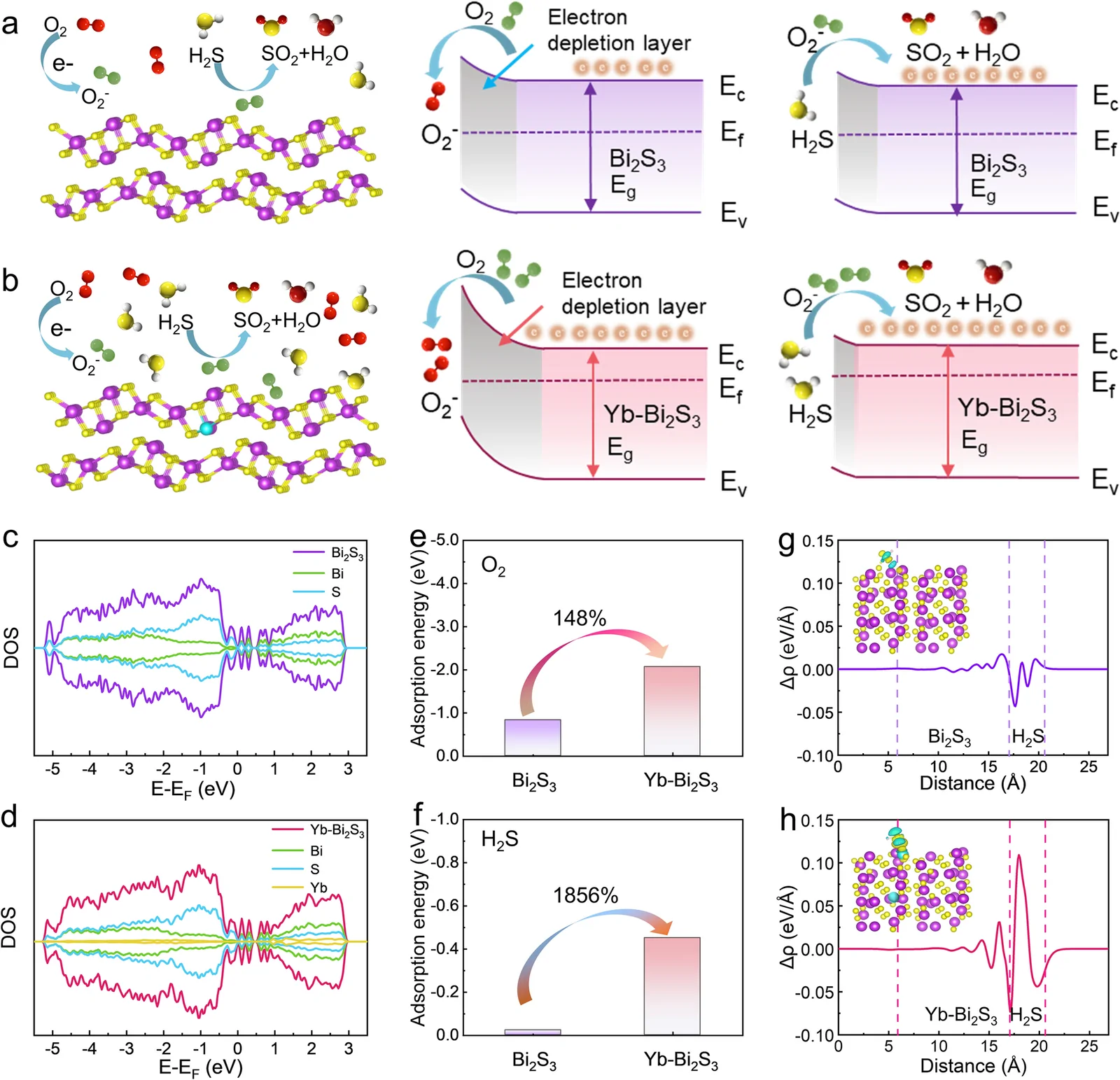
Scheme of the gas sensing mechanism and DFT calculations of the sensitive materials. Schematic of the H_2_S sensing process for **a** Bi_2_S_3_ and **b** Yb-Bi_2_S_3_. Density of states (DOS) for **c** Bi_2_S_3_ and **d** Yb-Bi_2_S_3_. DFT-calculated adsorption energy of Bi_2_S_3_ and Yb-Bi_2_S_3_ toward **e** O_2_ and **f** H_2_S. The charge density difference and corresponding one-dimensional charge density difference of **g** Bi_2_S_3_ and **h** Yb-Bi_2_S_3_

Previous results reveal the H_2_S sensing mechanism based on pure Bi_2_S_3_ is governed by surface redox reactions involving adsorbed oxygen species [39]. Herein, the Yb doping modifies the electronic structure and surface chemistry, thereby enhancing adsorption–reaction behavior and depletion layer modulation compared with pure Bi_2_S_3_. For pure Bi_2_S_3_ (Fig. 5a), oxygen molecules adsorb on the surface in air and capture conduction band electrons to form O_2_⁻ species (Eqs. (1) and (2)), generating a thin electron depletion layer and establishing baseline resistance [48, 49]. Upon exposure to H_2_S, these oxygen species react with the target gas and release electrons back into the material, reducing the depletion-layer width and lowering the resistance (Eq. (3)) [50]. In our experiments, Bi_2_S_3_ derived from CAU-17 exhibits a rich porous structure and large specific surface area, facilitating the adsorption of target gases. However, pure Bi_2_S_3_ possesses inherently limited active sites and activation capacity. Consequently, fewer oxygen species and H_2_S molecules participate in the reaction, resulting in weaker sensitivity and sluggish response/recovery behavior[24].

1 $${\mathrm{O}}_{2(gas)} \to {\text{ O}}_{2(abs)}$$

2 $${\mathrm{O}}_{{2(gas)}} + e^{ - } \to {\mathrm{O}}_{{2(abs)}}^{ - }$$

3 $$2{\mathrm{H}}_{2} {\mathrm{S}}_{{(gas)}} + 3{\mathrm{O}}_{{2(abs)}}^{ - } + e^{ - } \to 2{\mathrm{H}}_{2} {\mathrm{O}}_{{(abs)}} + 2{\mathrm{SO}}_{2} + 3e^{ - }$$

After Yb doping (Fig. 5b), the gas-sensing behavior of Yb-Bi_2_S_3_ is markedly modulated. Firstly, the high charge density of Yb^3^⁺ enhances the adsorption affinity for polar O_2_ molecules, leading to the formation of abundant O_2_⁻ adsorbed oxygen species on the Yb-Bi_2_S_3_ surface under ambient air conditions. The thickness of the corresponding electron depletion layer increases significantly, providing richer active species for subsequent H_2_S reactions [51]. Secondly, the mismatch in ionic radii between Yb^3^⁺ and Bi^3^⁺ induces lattice distortion, thereby generating numerous surface defect sites (sulfur vacancies) [52, 53]. These sites can serve as additional active sites to promote the adsorption of oxygen species and H_2_S. Concurrently, the unique 4*f* electronic states of Yb regulate the electron cloud distribution of Bi-S bonds, reducing the activation energy barrier for H_2_S-adsorbed oxygen reactions and substantially accelerating redox reaction rates. Thirdly, Yb doping optimizes the surface adsorption–desorption kinetics of the material. H_2_S rapidly reacts with abundant adsorbed oxygen, and the reaction products can quickly desorb from the surface. Air can then re-adsorb O_2_ to form adsorbed oxygen species, enabling the depletion layer to rapidly return to its initial state and shortening the sensor recovery time effectively.

To clarify how Yb doping modulates the sensing behavior of Bi_2_S_3_ at the atomic–electronic level, DFT calculations were conducted to analyze the density of states (DOS), gas adsorption energies, and interfacial charge transfer. Figure 5c, d present the total and element-resolved DOS of pure Bi_2_S_3_ and Yb-Bi_2_S_3_, respectively. In pure Bi_2_S_3_ (Fig. 5c), the electron density below the Fermi level (E_F_) is primarily contributed by the S orbitals, while the density above the E_F_ is mainly provided by the Bi elements. Following Yb doping (Fig. 5d), the 4*f* states of Yb hybridize with Bi and S states near E_F_. This enhanced orbital coupling increases electron density near E_F_ and reduces the bandgap, thereby providing a more favorable electronic structure for gas adsorption and charge transfer processes [35]. These theoretical results are in full agreement with those obtained from UPS and UV–Vis spectroscopy, specifically the decrease in the work function from 4.91 to 4.57 eV and the decrease in the bandgap from 1.35 to 1.12 eV. This optimization of the electronic structure accelerates the kinetics of surface redox reactions, as evidenced by a reduction in response time from approximately 25 s for pure Bi_2_S_3_ to 11 s for Yb-Bi_2_S_3_. Furthermore, compared to Bi, Yb provides more vacant orbitals, effectively enhancing the adsorption of electron-rich substances like O_2_ and H_2_S and improving sensing sensitivity. In addition, the adsorption energy calculation results in Fig. 5e, f show that Yb-Bi_2_S_3_ exhibits a 148% higher adsorption energy for O_2_ compared to pure Bi_2_S_3_, along with a prominent 1856% improvement in H_2_S adsorption. These theoretical calculations confirm that Yb doping greatly strengthens the adsorption capability of the material toward O_2_ and H_2_S. Strong O_2_ adsorption provides ample active species for oxygen-dominated redox reactions, while robust H_2_S adsorption ensures efficient interaction between target molecules and the sensing surface [54, 55]. Together, these enhanced adsorption energies establish the solid foundation for the finding that the response value of Yb-Bi_2_S_3_-1.5% at a H_2_S concentration of 100 ppb (3.31) is significantly higher than that of pure Bi_2_S_3_ (~ 1.5).

To verify the charge transfer interaction between Bi_2_S_3_ and Yb-Bi_2_S_3_ with H_2_S, the charge density difference and corresponding one-dimensional charge density difference results are shown in Fig. 5g, h. After H_2_S adsorbed on Yb-Bi_2_S_3_, the charge distribution disturbance between H_2_S and the material surface is significantly greater than that of pure Bi_2_S_3_, revealing more intensive interfacial charge transfer. Within the H_2_S molecule, a distinct charge transfer occurs between H and S, facilitating H–S bond cleavage and thereby promoting H_2_S oxidation. This indicates that Yb doping enhances the charge transfer effect during the adsorption process. Combined with Bader charge analysis (Fig. S13), during H_2_S adsorption on pure Bi_2_S_3_, for H_2_S adsorbed on pure Bi_2_S_3_, the calculated charge of S in H_2_S is 0.113 *e*, and that of H is −0.046 *e*. For the Yb-Bi_2_S_3_ system, the Yb atom carries a charge of −1.714 *e*; meanwhile, the charge of S in adsorbed H_2_S increases to 0.196 *e*, while the charge of H decreases to −0.078 e.The above results indicate that, on one hand, the covalent nature of H_2_S molecules adsorbed on Yb-Bi_2_S_3_ weakens, favoring H–S bond cleavage and providing favorable conditions for subsequent oxidation reactions [56]. On the other hand, H_2_S molecules on the Yb-Bi_2_S_3_ surface acquire a greater number of electrons, resulting in stronger interactions between the two [57]. Accordingly, Yb introduction greatly promotes charge transfer during H_2_S adsorption via an enhanced electron-withdrawing capability. Such promoted charge transfer further enlarges the resistance variation of the sensing material, endowing Yb-Bi_2_S_3_ with improved gas sensing sensitivity of 0.02/ppb (20/ppm) in the H_2_S concentration range of 5–200 ppb.

### Periodontitis Diagnosis via Oral Exhalation H_2_S Sensor

**Fig. 6:**
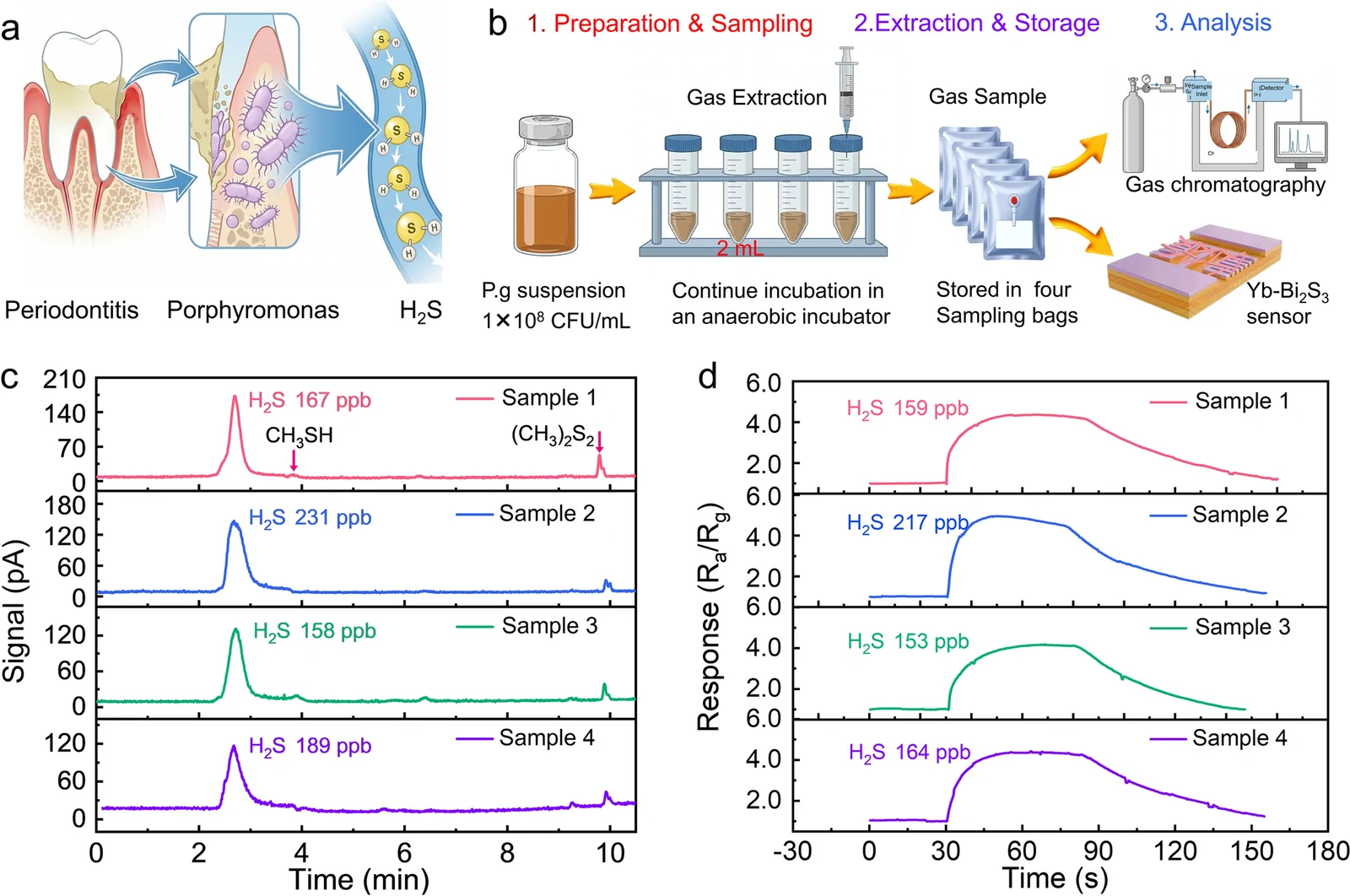
Yb-Bi_2_S_3_ sensor validates *p.g.*-mediated H_2_S production in periodontitis. **a** Schematic illustration of H_2_S production by *p.g.* in the oral cavity of periodontitis patients. **b** Schematic diagram of the sampling, storage, and analysis procedures for H_2_S generated by *p.g*. **c** Gas chromatography and **d** Yb-Bi_2_S_3_ sensor determination of H_2_S concentration generated by *p.g.*

Pathogenic bacteria in the oral cavity of periodontitis patients may produce H_2_S, yet the relationship between periodontitis, pathogens, and endogenous H_2_S levels remains poorly elucidated. Traditional H_2_S detection mainly relied on chromatography, with limitations of complex operation and high cost [33]. In this regard, the Yb-Bi_2_S_3_ sensor provides a facile alternative to explore such biological relevance. *Porphyromonas gingivalis* (*p.g.*), a key H_2_S-producing pathogen in periodontitis, predominantly colonizes the subgingival biofilm of periodontal pockets (Fig. 6a) [8, 58]. It decomposes host-derived sulfur-containing amino acids via the cystathionine biosynthetic pathway to produce H_2_S [31, 59]. Therefore, *p.g.* was selected as the model to validate the association between periodontitis pathogens and H_2_S emission using the Yb-Bi_2_S_3_ sensor. In brief, the suspension of *p.g.* was aliquoted into 4 × 2 mL portions and sealed in four 15 mL conical tubes (Fig. 6b, see Experimental Section for details). The volatile gases above the bacterial suspension were extracted with a syringe and stored in 10 mL gas sampling bags. The obtained gas samples were quantitatively analyzed using GC and the Yb-Bi_2_S_3_ sensor, respectively. The GC results reveal that* p.g.* produces H_2_S, CH_3_SH, and (CH_3_)_2_S_2_, with H_2_S as the dominant gas (Fig. 6c). The H_2_S concentrations measured by GC in four samples were 167, 231, 158, and 189 ppb, respectively. For Yb-Bi_2_S_3_ sensor measurement (Fig. 6d), the sensor was placed in a 5 mL airtight test chamber with external electrical leads. Testing the same four gas samples yielded H_2_S concentrations of 159, 217, 153, and 164 ppb, respectively. The H_2_S concentrations detected by the Yb-Bi_2_S_3_ sensor were in good accordance with GC data, with relative deviations of 4.79%, 6.06%, 3.16%, and 13.22%, respectively. Although minor detection errors existed, sensor sensitivity could be further improved through calibration and correction. These results confirm that *p.g. *is a major H_2_S-producing pathogen in periodontitis patients and provide quantitative references for the correlation between bacterial concentration and H_2_S production.

**Fig. 7:**
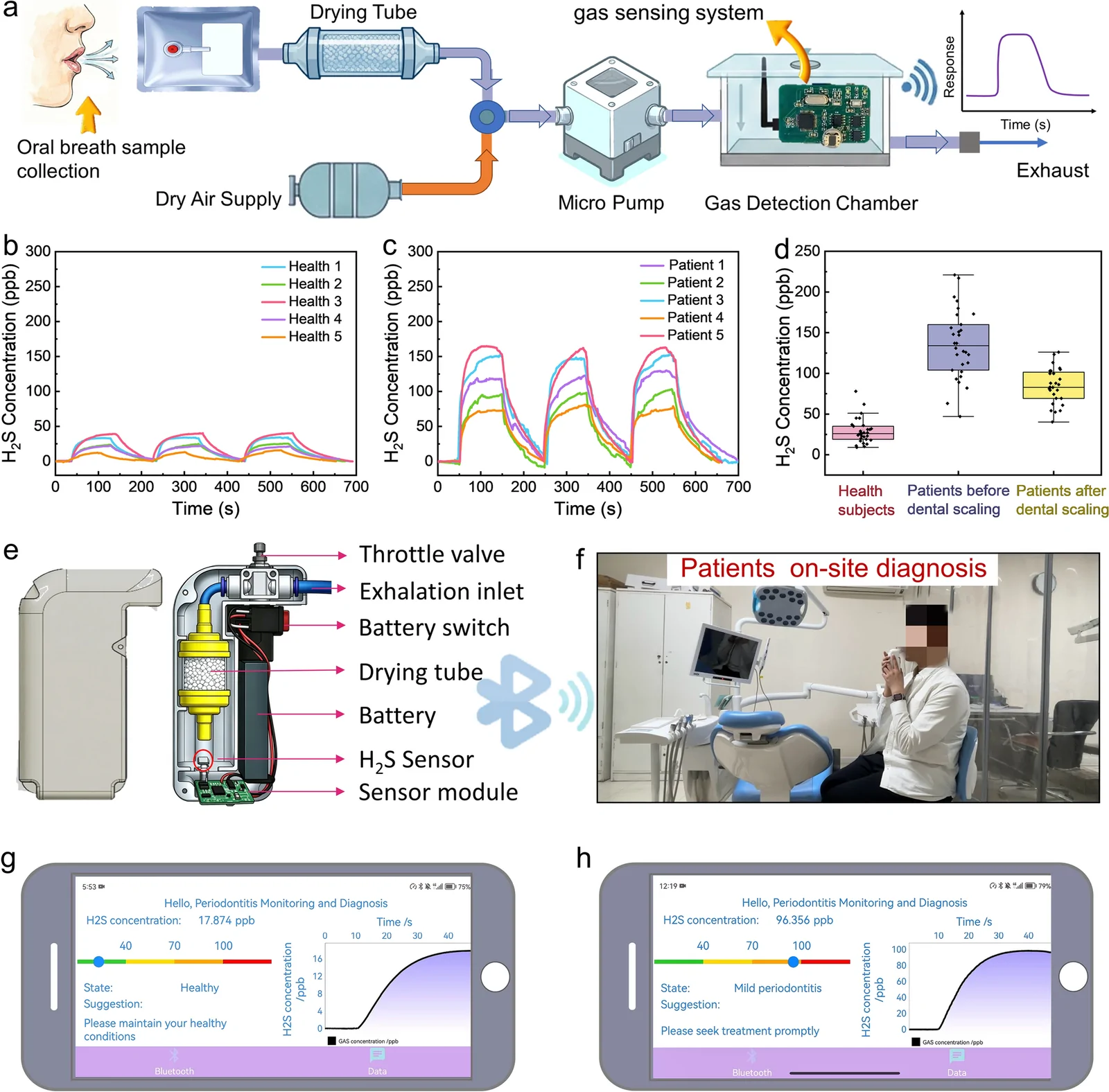
Application validation of Yb-Bi_2_S_3_ sensors for Oral H_2_S detection in periodontitis. **a** Schematic of the flowchart for H_2_S detection in oral exhalation. **b** H_2_S concentration curves in oral exhalation of healthy subjects. **c** H_2_S concentration curves in oral exhalation of periodontitis patients (pre-dental scaling). **d** Statistical distribution of exhaled H_2_S concentrations in healthy subjects and periodontitis patients (pre- and post-dental scaling). **e** Internal structure diagram of the periodontitis analyzer. **f** Photograph of a periodontitis patient undergoing diagnosis with the periodontitis analyzer at the hospital. Smartphone interface for displaying H_2_S detection results from the periodontitis analyzer for **g** a healthy subject and **h** a periodontitis patient

To validate the sensor’s applicability for diagnosing and monitoring periodontitis, the Yb-Bi_2_S_3_ sensor was integrated into a circuit system to form a gas detection module. Practical validation was conducted using exhaled samples from healthy individuals and hospital-diagnosed periodontitis patients (moderate/severe, pre- and post-dental scaling) (Fig. 7a, detailed sampling procedures are provided in the Experimental Section). The collected exhalation samples were first dried to eliminate humidity interference. A small pump connected to a three-way valve at the drying tube outlet dynamically delivered exhaled breath into a 200 mL detection chamber for gas module analysis, with chamber purging via air pathway during exhalation recovery. Within the gas module, the sensor’s response is converted into gas concentration output for more intuitive diagnosis. Five healthy individuals and five periodontitis patients (before and after gingival scaling) were randomly selected from the volunteers, and their oral exhalation samples underwent three replicate tests. Consistent sensing outputs were observed in healthy subjects, with measured H_2_S concentrations ranging from 12 to 39 ppb (Fig. 7b). For periodontitis patients, stable sensing signals were also noted, with pre-dental scaling H_2_S concentrations of 73–164 ppb (Fig. 7c). Post-dental scaling, exhaled H_2_S concentrations in periodontitis patients decreased significantly, and some returned to levels comparable to healthy individuals (Fig. S14). These results clearly demonstrate the sensor’s capability to reliably discriminate oral H_2_S levels between healthy individuals and periodontitis patients. In addition, the variability of three repeated tests was analyzed for the aforementioned exhaled samples. Statistical analysis revealed that the standard deviation (SD) of the triplicate measurements ranged from 2.61% to 12.4% for healthy individuals, and from 1.22% to 4.68% (pre-scaling) and 1.34%–6.82% (post-scaling) for periodontitis patients. The variation in H_2_S concentrations measured in the exhaled samples of all subjects across three tests remained at a low level, highlighting the high repeatability and reliability of the sensor in the practical detection of periodontitis. To further confirm gas sensing system reliability, additional exhalation samples were collected from 30 healthy individuals and 30 periodontitis patients (before and after dental scaling) for single tests. Statistical analysis was performed on the exhaled gas detection values (Fig. 7d). The data are presented as mean ± standard deviation (SD). Comparisons between healthy individuals and periodontitis patients were analyzed using an independent-samples t-test, whereas the comparison of periodontitis patients before and after dental scaling was analyzed using a paired-samples t-test. The results show significantly higher measured H_2_S concentrations in periodontitis patients compared to the healthy group, at 135.5 ± 42.3 ppb and 28.8 ± 15.37 ppb, respectively. This is consistent with previous studies reporting oral H_2_S concentrations of 100–150 ppb or higher in periodontal disease patients [4]. Post-dental scaling, the concentration of H_2_S in the exhaled breath of patients with periodontitis decreased significantly to 84.28 ± 21.70 ppb, with some patients’ exhaled H_2_S concentrations returning to levels seen in healthy individuals. The decrease in H_2_S concentration in patients with periodontitis following scaling is attributed to the physical removal of dental plaque and pathogenic bacteria, which substantially reduces the bacterial reservoir responsible for H_2_S production. And the reduction in H_2_S following professional scaling combined with oral hygiene instruction is sustained for several weeks to several months [60, 61]. In contrast, the suppression of H_2_S after daily tooth brushing is short‑lived because brushing alone does not eliminate the deep subgingival pathogenic flora [62]. Besides, high standard deviations in exhaled H_2_S were attributed to individual variations and oral environment differences. Although strict standardization procedures were implemented prior to sampling, differences in oral microbiome composition, salivary flow, and tongue coating among individuals can also influence gas levels in the oral cavity. Furthermore, there are variations in the severity and spatial distribution of periodontal lesions, including periodontal pocket depth, inflammation levels, and bacterial colonization, which directly contribute to a wider range of variation in patients’ exhaled H_2_S levels. Importantly, the substantial separation between group means confirms that these variances do not compromise diagnostic discrimination. In future clinical diagnostics, it will also be necessary to further standardize oral conditions in order to maximize the detection accuracy of the sensor modules.

To validate the potential of the sensor module for direct real-time diagnosis in clinical and community hospitals, it has been integrated into a periodontitis analyzer. The device comprises a battery, a switch, an exhalation port, an exhalation throttle valve, a drying tube, and a sensor module (Fig. 7e). Participants exhale through the exhalation port, and collected test data are wirelessly transmitted to a smartphone via Bluetooth for real-time display and quantitative analysis (Fig. 7f). Notably, a drying tube filled with anhydrous calcium chloride is installed upstream of the sensor to eliminate moisture interference. This drying tube achieves a dehumidification rate of approximately 90% without affecting H_2_S concentrations. Moreover, the desiccant cartridge can be routinely replaced after a fixed number of tests to maintain stable dehumidification capacity, making the device suitable for large-scale population screening and household self-monitoring.The actual exhalation testing process using this periodontitis analyzer for 3 healthy individuals and 3 periodontitis patients is demonstrated in Movies S1 and S2. According to standard operating procedures, all participants remained seated at rest with closed mouths and nasal breathing for 3 min to stabilize oral H_2_S concentration and reach gas equilibrium. Meanwhile, the periodontitis analyzer was powered on and Bluetooth-paired with a smartphone to enter standby detection mode. In the formal test, each subject performed slow, continuous exhalation for 40–50 s to guarantee a stable sensor signal output. The device rapidly responds upon effective exhalation and maintains a stable final reading. As representative cases, the detected oral H_2_S concentration of the healthy volunteer was 17.87 ppb (Fig. 7g), while the periodontitis patient exhibited a much higher value of 96.35 ppb. Correspondingly, the supporting software automatically labeled a mild periodontitis risk and provided targeted health guidance (Fig. 7h). These typical cases verify that the portable analyzer can effectively differentiate exhaled H_2_S features between healthy and periodontitis populations, demonstrating great potential as a convenient, portable, and user-friendly tool for daily oral health surveillance. For practical breath analysis, device safety and reusability are also fully considered. The integrated sensor module works entirely at room temperature and requires no auxiliary heating units, which is highly favorable for non-invasive oral breath detection. Disposable mouthpieces are adopted for individual use to avoid cross-contamination and ensure clinical hygiene. Benefiting from the modular design, the desiccant tube and sensor chip can be conveniently replaced on-site after long-term operation. Collectively, these structural and functional advantages guarantee the excellent safety, long-term stability, and satisfactory reusability of the developed periodontitis detection device.

## Conclusion

In summary, this study develops a Yb-Bi_2_S_3_-based H_2_S sensor for the point-of-care diagnosis of periodontitis. The sensor exhibits a wide linear response range (5 ppb to 5 ppm), high response (3.31 at 100 ppb), fast response kinetics, and excellent selectivity under ambient temperature conditions. DFT calculations verify that the superior performance stems from Yb doping-induced lattice distortion, which promotes oxygen adsorption and facilitates H_2_S-specific binding. Validations based on periodontal pathogens, clinical exhaled breath samples, and on-site human testing establish a quantitative correlation between *Porphyromonas gingivalis* proliferation and breath H_2_S levels, allowing effective discrimination between healthy individuals and periodontitis patients. For clinical translation, variability from individual oral physiology, dietary sulfur intake, and suboptimal oral hygiene necessitates further standardized sampling protocols and periodic sensor calibration. Nonetheless, the portable analyzer holds considerable promise for home self‑monitoring, large‑scale community screening, and objective post‑treatment surveillance. Featuring non-invasive and on-site detection, this device lays a solid foundation for the development of intelligent oral healthcare diagnostic systems.

## Supplementary Information

Below is the link to the electronic supplementary material.

Supplementary file1 (MP4 3996 KB)

Supplementary file2 (MP4 3915 KB)

Supplementary file3 (DOCX 4084 KB)
