# Supplementary material for: On-Site Periodontitis Diagnosis via Room-Temperature Oral Exhalation H2S Sensor Based on Yb-Doped Bi2S3 Nanoribbon
Source: Nanomicro Lett. 2026 Jul 8;18:430. doi: 10.1007/s40820-026-02273-x (PMC13346398; doi:10.1007/s40820-026-02273-x)
Supplement: Supplementary file 3 — Supplementary file3 (DOCX 4084 KB) [file 40820_2026_2273_MOESM3_ESM.docx]

# Supporting Information for

**On-Site Periodontitis Diagnosis via Room-Temperature Oral** **Exhalation H_2_S Sensor Based on Yb-Doped Bi₂S₃ Nanoribbon**

Ping Guo ^1^, Kai Wang ^2^, Xuyang An ^1^, Xuanyu Ren ^3^, Xinxin He ^1^, Yinhua Hu ^1^, Lijie Zhou ^4^, Guohua Cai ^5^, Lifeng Wang ^6^, Tiezhu Liu ^1^*, Wenyu Ge ^2^*, Jia Zhang^1, 7^*

^1^ State Key Laboratory of Robotics and System, Harbin Institute of Technology, Harbin 150080, P. R. China

^2^ Department of Stomatology Center, Heilongjiang Provincial Hospital, Harbin 150036, P. R. China

^3^ Key Laboratory of Bio-Based Material Science and Technology of the Ministry of Education, Northeast Forestry University, Harbin150040, P. R. China

^4^ School of Mechanical and Power Engineering, Harbin University of Science and Technology, Harbin, 150080, P. R. China

^5^ The Second Affiliated Hospital of Harbin Medical University, Harbin, 150081, P. R. China

^6^ School of Science, City Campus Australia, RMIT University, 124 La Trobe Street, Melbourne VIC 3000, Australia

^7^Key Laboratory of Micro-Systems and Micro-Structures Manufacturing, Ministry of Education, Harbin Institute of Technology, Harbin 150080, P. R. China

*Corresponding authors. E-mail:  [zhangjia@hit.edu.cn](mailto:zhangjia@hit.edu.cn) (Jia Zhang); [liutiezhu@hit.edu.cn](mailto:liutiezhu@hit.edu.cn) (Tiezhu Liu); [gwysci@163.com](mailto:gwysci@163.com) (Wenyu Ge)

**Supplementary Figures and Tables**


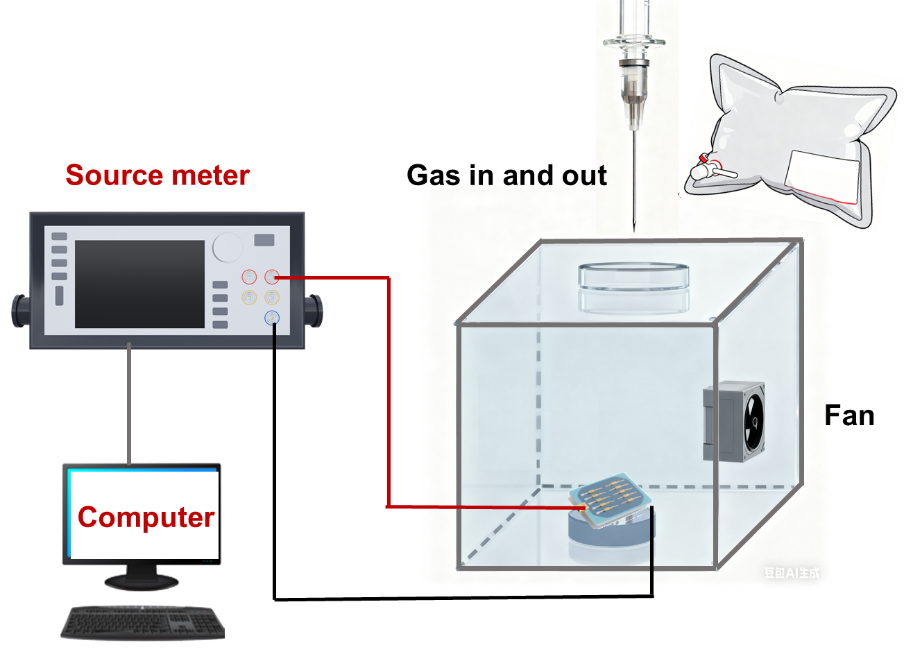


**Fig. S1** Schematic diagram of the gas sensing test system.


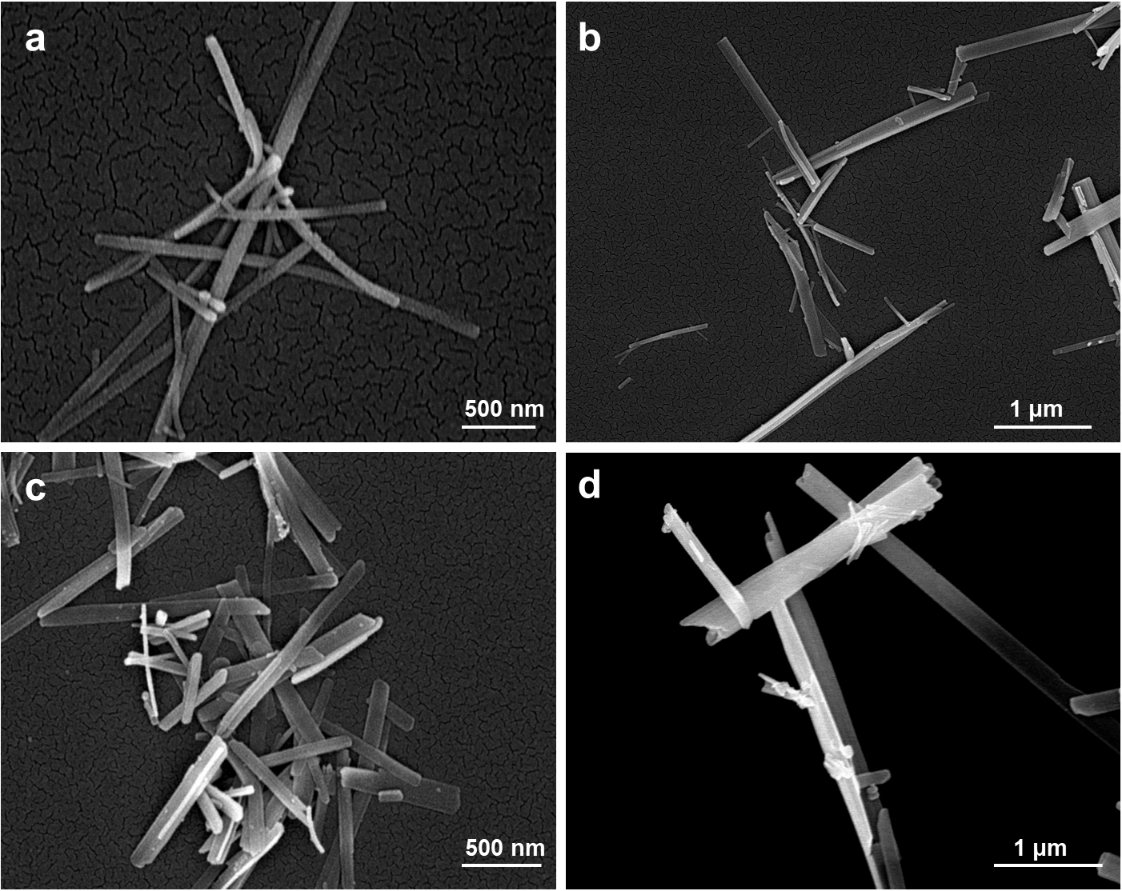


**Fig. S2** SEM images of (a) Yb-Bi_2_S_3_-0.2%, (b) Yb-Bi_2_S_3_-0.5%, (c) Yb-Bi_2_S_3_-1.0 %, (d) Yb-Bi_2_S_3_-2.0%.


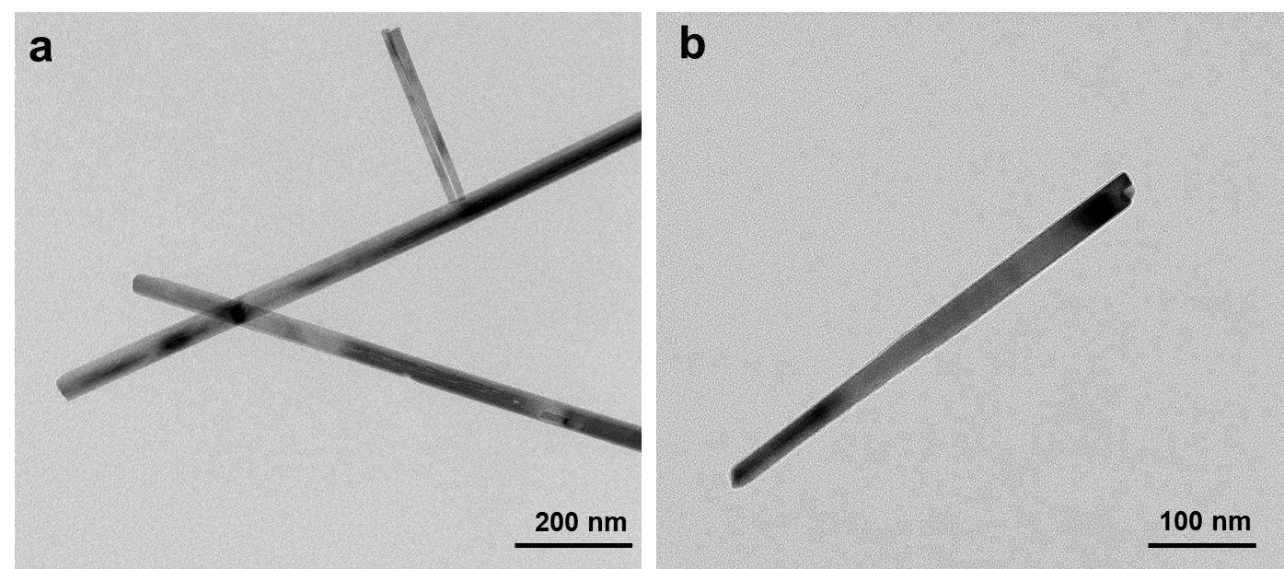


**Fig. S3** TEM images of Yb-Bi₂S₃-1.5%.

******

**Fig.S4** Comparison of high-resolution C 1s spectra between Yb-Bi₂S₃ derived from CAU-17 and Yb-Bi₂S₃ synthesized by a conventional one-step hydrothermal method.

**
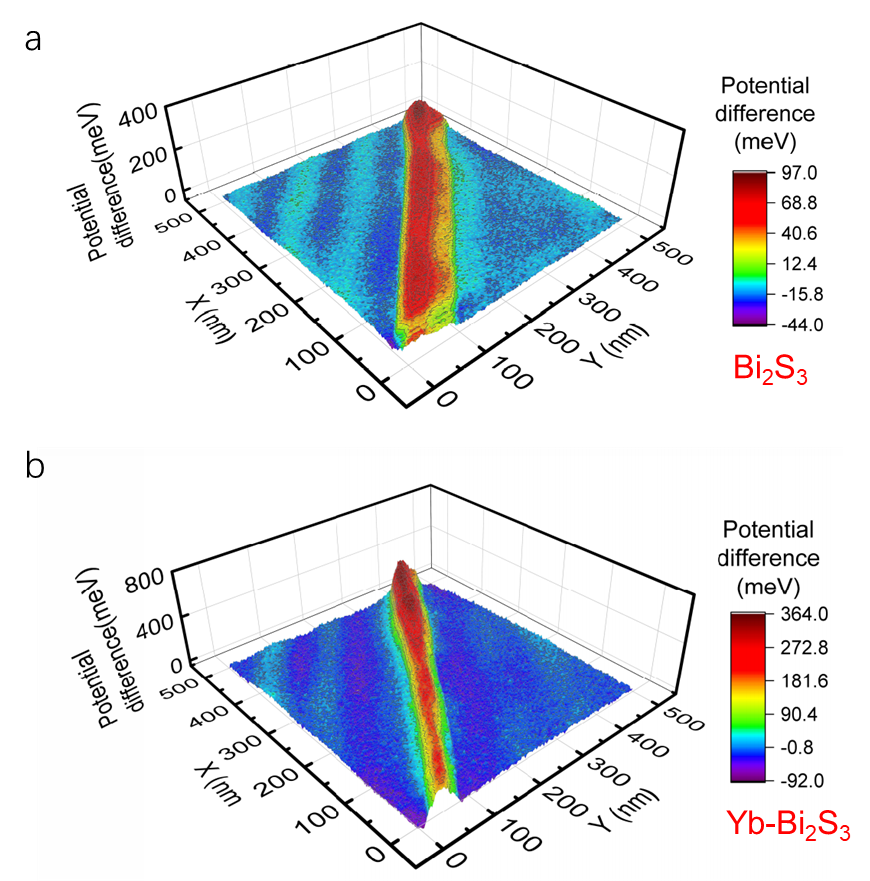
**

**Fig. S5** Work functions measured by Kelvin probe force microscopy (KPFM). (The relationship between the contact potential difference (CPD) and the work function (Φ) is given by CPD _sample_ =CPD _Au_+(Φ _Au_−Φ _sample_), where CPD _Au_=−0.18, and Φ _Au_=5.10 eV. The measured CPD values for the Bi_2_S_3_ and Yb-Bi_2_S_3_ were approximately 0.08 eV and 0.34 eV, yielding corresponding work functions of 4.84 eV and 4.59 eV, respectively.)


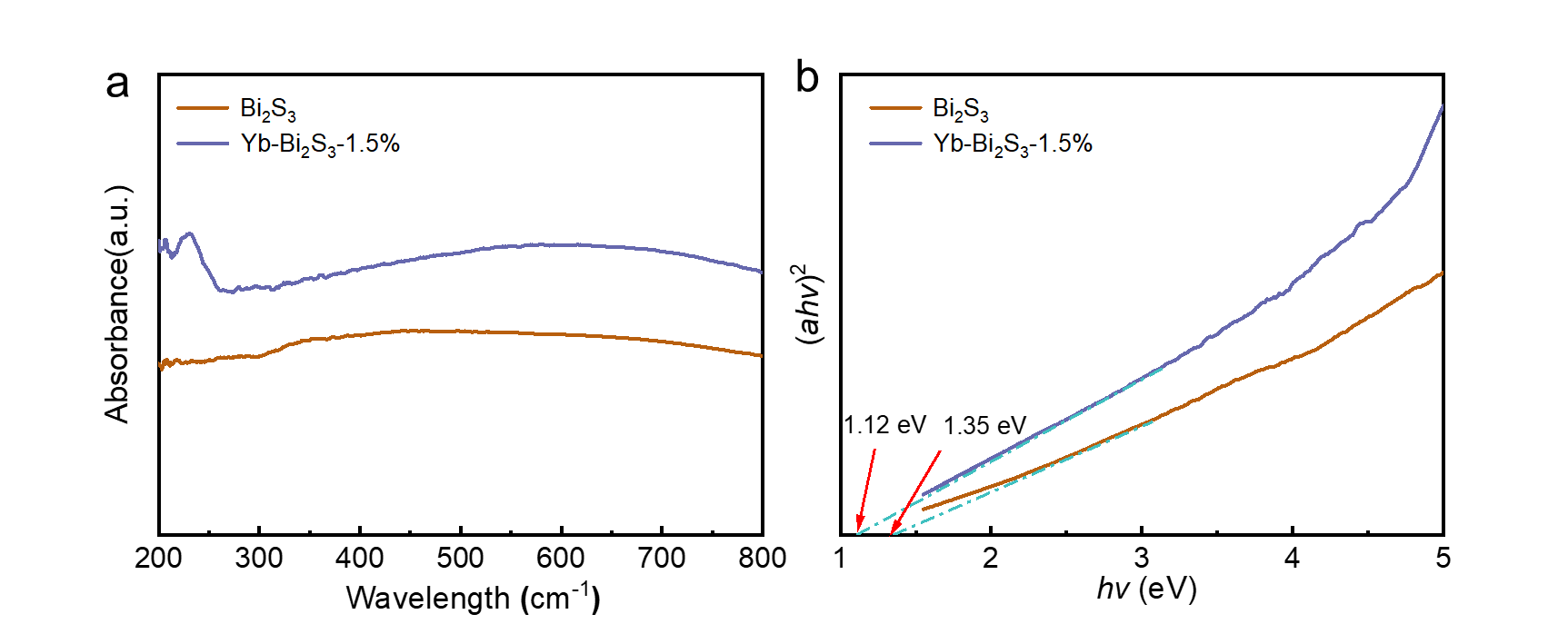


**Fig.S6** The optical properties of BS and Yb-Bi₂S₃-1.5%. (a) UV–vis spectra. (b) the calculated band gap.

**Fig. S7** The response curve of Yb-Bi₂S₃-1.5% to 5 ppb H_2_S.

**Fig. S8** A single-range fitting of the response as a function of concentration within the range of 0.005–5 ppm.


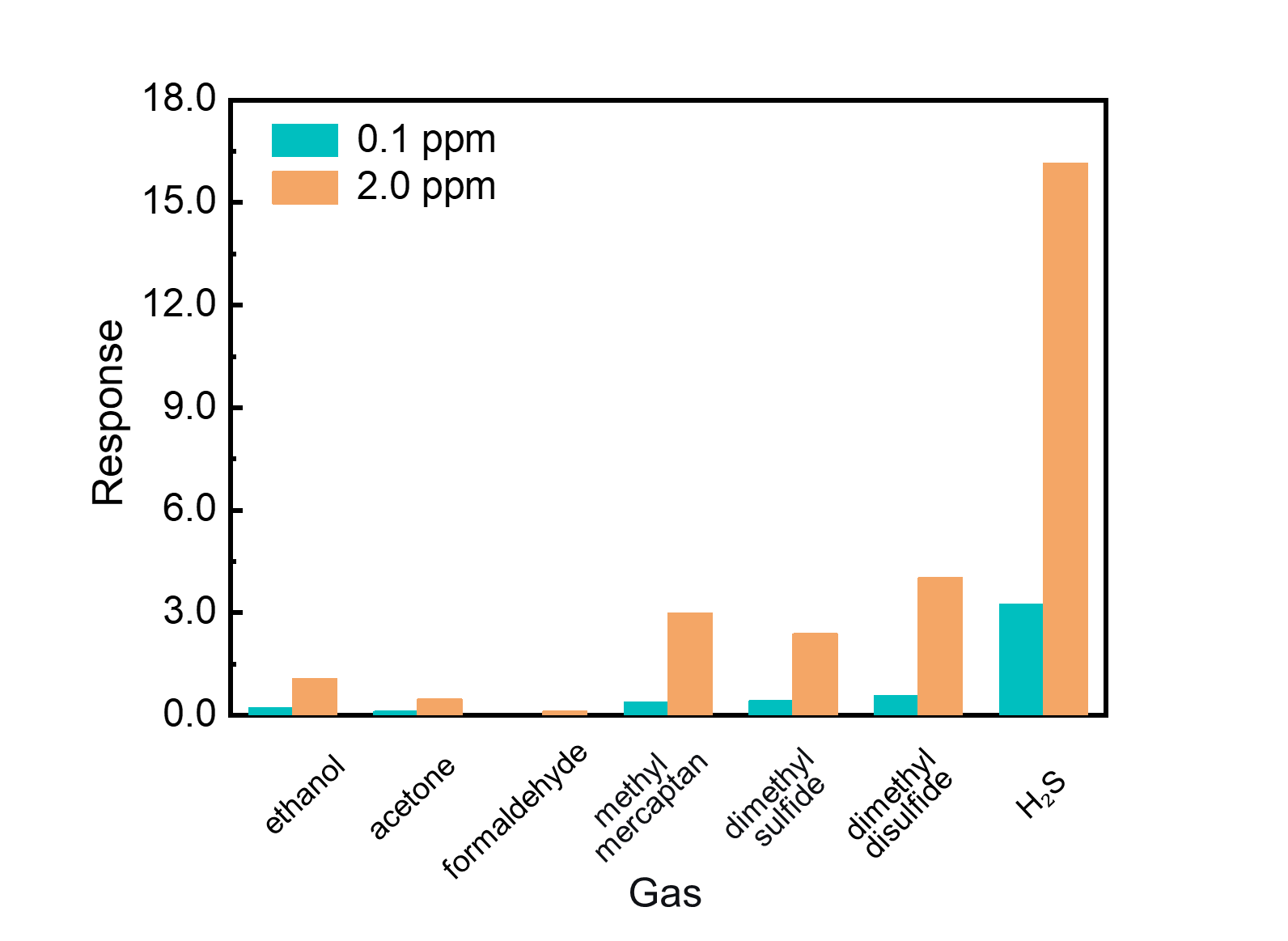


**Fig. S9** Selective response of Yb-doped Bi₂S₃ sensor toward VOCs and VSCs


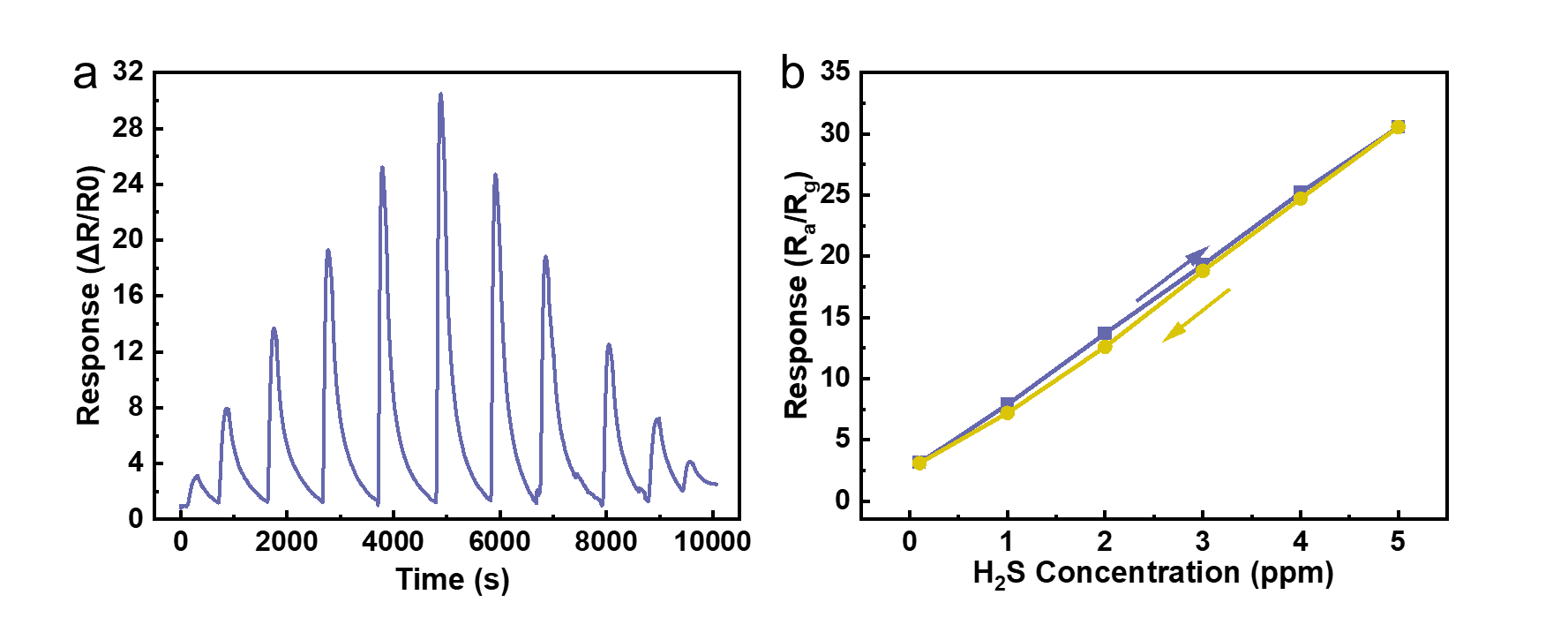


**Fig. S10** Hysteresis characteristic of Yb-Bi₂S₃-1.5% in the range of 0.1-5 ppm.

**Fig. S11** Repeatability test of Yb-Bi₂S₃-1.5% for Repeated exhalation exposure. (A specific volume of H₂S standard gas was mixed with high-humidity air to prepare simulated oral breath containing 100 ppb H₂S for testing. A dynamic testing method was employed: the gas in the sample bag was first dried to eliminate humidity interference. A small pump connected to a three-way valve at the outlet of the drying tube dynamically delivered gas from the gas bag to a 200-mL detection chamber for gas sensing.)


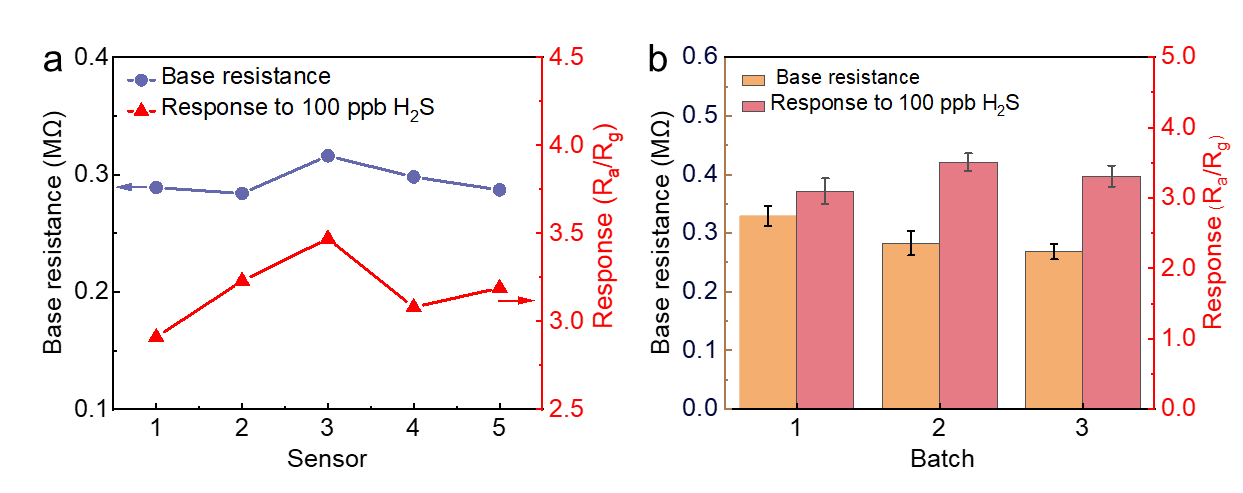


**Fig. S12** (a) Baseline resistance and response to 100 ppb H₂S for five sensors from the same batch; (b) baseline resistance and response to 100 ppb H₂S for sensors from different batches


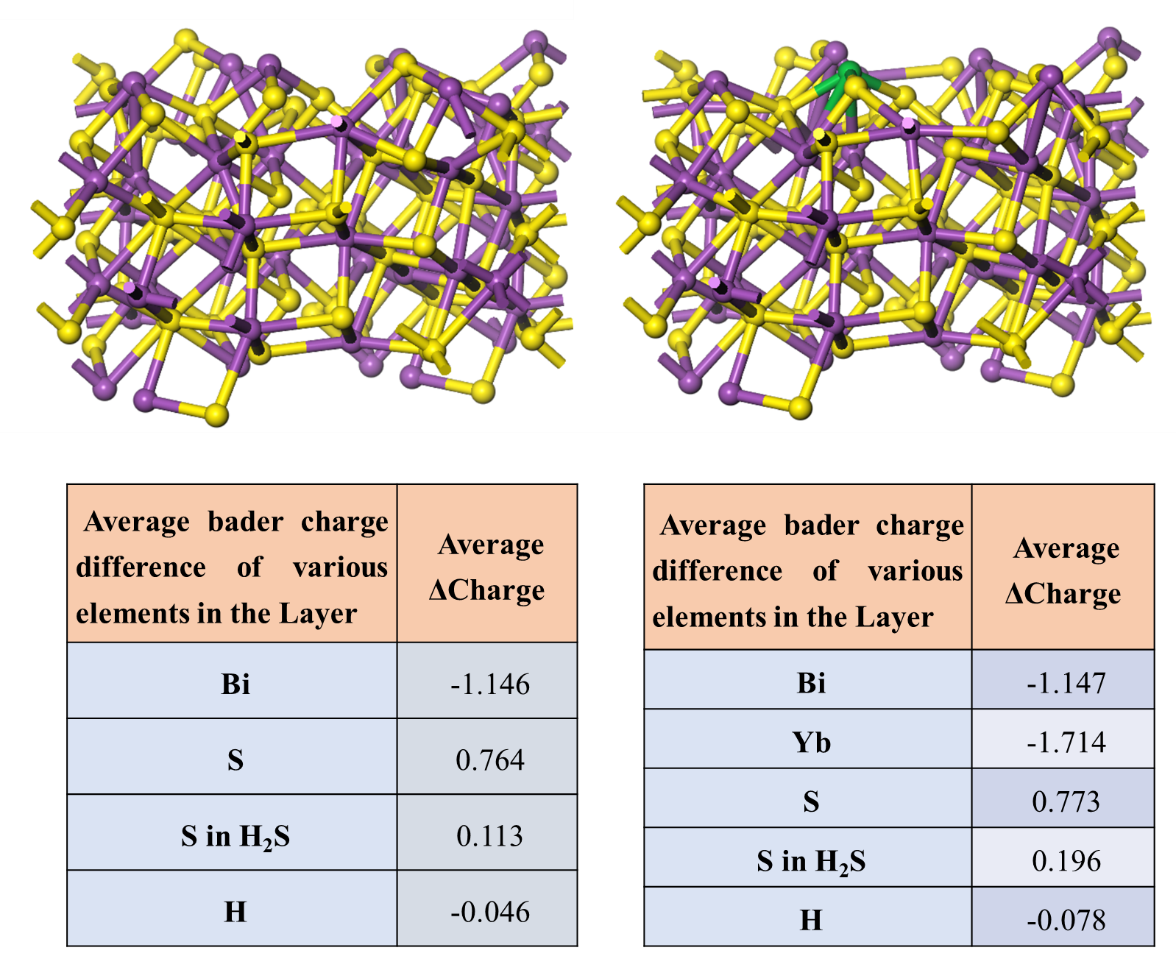


**Fig. S13** Bader charge of H_2_S adsorption on the surface of BS and Yb-Bi₂S₃-1.5%, respectively.

**Fig. S14** H₂S concentration curves in oral exhalation of periodontitis patients (post-dental prophylaxis).

**Table S1** A comparative table of latest representative H₂S sensor based on different sensitive material

| **Materials** | **Operating temperature** | **Detection limit(ppb)** | **Response** | **t_res_/t_rec_** | **Detection**  **range** | **Humidity**  **tolerance** | **Selectivity** | **Sensor Integration** | **Ref.** |  |
| --- | --- | --- | --- | --- | --- | --- | --- | --- | --- | --- |
| Metal Oxides and Heterojunctions | | | | | | | | | | |
| Amorphous a-SnO_2_ | 100 ℃ | 210 ppb | 2.4 (1ppm） | 9 min/- | 400 ppb ~ 1.5 ppm | ~20% reduction | - | no | [1] |  |
| Rh_2_O_3_/ WO_3_ | 157℃ | 250 ppb | ~5.2 (1 ppm) | 12/64 s | 0.25-100 ppm | ~75% reduction | > 22 | no | [2] |  |
| Bi_2_MoO_6_–CuO | 180 °C | 5 ppb | 4.9 (0.1 ppm) | 213s/1069s | 5-250 ppb |  | > 7 | yes | [3] |  |
| α-Fe_2_O_3_/SnO_2_ | 250 °C | 1 ppm | 4.3 (10 ppm) | 13s/104s | 2–10 ppm | - | >3 | no | [4] |  |
| Co_3_O_4_/β-CoMoO_4_ | 160 °C | 10 ppb | 1.25 (10 ppb) | 288/731 s | 0.01–20 ppm | - | > 2 | yes | [5] |  |
| Cu₂O/NiO | RT | 10 ppm | 1.39 (20 ppm) | ~100 s | 10–100 ppm | - | >10 | no | [6] |  |
| In_2_O_3_/SnO_2_ | RT | 100 ppb | ~5.2 (0.1 ppm) | 600/181 s | 0.1-70 ppm | ~53% reduction | > 15 | yes | [7] |  |
| WO_3_/CuO | 150℃ | 0.5 ppm | 0.68 (5ppm） | 24 s / 78 s | 0.5–50 ppm | - | >6 | no | [8] |  |
| Fe_2_O_3/_Ti_3_C_2_ | RT | 10 ppb | 1.18 (20 ppm) | 10s/15s | 0.01–500 ppm | ~28% reduction | >6 | no | [9] |  |
| Transition Metal Sulfides and Heterojunctions | | | | | | | | | | |
| WS_2_ | RT | 53 ppb | 0.15 (27 ppm) | 175 s/- | 53 ppb-27 ppm | ~400% increase | > 10 | no | [10] |  |
| Bi_2_S_3_ | RT | 0.5 ppm | 2.03 (10 ppm) | 27 s/21 s | 0.5–50 ppm | - | > 10 | no | [11] |  |
| MoSe_2_/ZnO | 150 ℃ | 200 ppb | 2.8 (6 ppm) | 690 s/665 s | 200 ppb–6.1 ppm | - | ~2 | no | [12] |  |
| MoS_2_/rGO | RT | 1 ppm | 1.18 (1ppm) | 0.85/12.1 s | 1-100 ppm | ~46% reduction | >5 | no | [13] |  |
| WS_2_- ZnO | 250 °C | 0.1 ppm | 0.33 (1ppm) | ~120s/ | 0.1-5 ppm | - | >8 | no | [14] |  |
| ZIF-L/SnS_2_ | 125 ℃ | 3.6 ppb | 1.11 (1 ppm) | 413s/2276s | 1-50 ppm | - | >10 | no | [15] |  |
| Other material systems | | | | | | | | | | |
| Cs_2_AgBiCl_6_ | RT | 5 ppb | 0.16 (0.08pm) | 99.6/94.2s | 5 ppb-10 ppm | - | >40 | yes | [16] |  |
| Co_3_(HITP)_2_/Ti_3_C_2_ | RT | 92 ppb | 2.41(10 ppm) | ~200 s/- | 0.1–200 ppm | ~12% reduction | >2 | no | [17] |  |
| CsPbBr_3_ | RT | <200 ppb | 1.21 (0.2 ppm) | 73/275 s | 0.2-5 ppm | <5% | >10 | no | [18] |  |
| This work | | | | | | | | | | |
| Yb-Doped Bi₂S₃ | RT | 5 ppb | 9.13 (1 ppm) | 11 s/141 s | 5 ppb-5 ppm | ~27% reduction | > 20 | yes | - |  |

**Supplementary References**

1. V. Paolucci, J. De Santis, V. Ricci, L. Lozzi, G. Giorgi et al., Bidimensional engineered amorphous *a*-SnO_2_ interfaces: synthesis and gas sensing response to H_2_S and humidity. ACS Sens. **7**(7), 2058–2068 (2022). <https://doi.org/10.1021/acssensors.2c00887>
2. D. Xu, R. Li, X. Duan, B. Sun, X. Gong et al., *In-situ* construction of highly sensitive H_2_S gas sensor based on Rh_2_O_3_-loaded WO_3_ composites derived from WSe_2_ flower-like structure. Sens. Actuat. B Chem. **460**, 139938 (2026). <https://doi.org/10.1016/j.snb.2026.139938>
3. Q. Jing, C. Gong, W. Bian, Q. Tian, Y. Zhang et al., Ultrasensitive chemiresistive gas sensor can diagnose asthma and monitor its severity by analyzing its biomarker H_2_S: an experimental, clinical, and theoretical study. ACS Sens. **7**(8), 2243–2252 (2022). <https://doi.org/10.1021/acssensors.2c00737>
4. X.-Y. Miao, L.-Y. Zhu, X.-Y. Wu, L.-W. Mao, X.-H. Jin et al., Precise preparation of α-Fe_2_O_3_/SnO_2_ core-shell nanowires *via* atomic layer deposition for selective MEMS-based H_2_S gas sensor. Sens. Actuat. B Chem. **378**, 133111 (2023). <https://doi.org/10.1016/j.snb.2022.133111>
5. S. Cao, Z. Song, Y. Bing, X. Xu, T. Zhou et al., Metal–organic-framework derived co–Mo multimetal oxide semiconductors: selective trace-level hydrogen sulfide detection. ACS Sens. **9**(6), 2979–2988 (2024). <https://doi.org/10.1021/acssensors.4c00144>
6. H. Sun, M. Cao, P. Zhang, X. Tian, M. Lu et al., Magnetic-field-enhanced H_2_S sensitivity of Cu_2_O/NiO heterostructure ordered nanoarrays. ACS Sens. **7**(7), 1903–1911 (2022). <https://doi.org/10.1021/acssensors.2c00495>
7. Y. Sun, K. Qian, J. Yue, X. Zhang, X. Li et al., High-response In_2_O_3_/SnO_2_ heterojunction sensor for detection of food spoilage gas H_2_S in refrigerators. Sens. Actuat. B Chem. **451**, 139341 (2026). <https://doi.org/10.1016/j.snb.2025.139341>
8. C. Qiu, L. Zhu, Q. Li et al., One-step electrospun WO_3_/CuO p–n heterojunction nanocomposites for ultrasensitive and rapid H_2_S detection. ACS Sens. **10**(9), 6869–6877 (2025). <https://doi.org/10.1021/acssensors.5c01761>
9. D. Zhang, X. Huang, W. Meng, J. Yuan, F. Guo et al., Room-temperature flexible CNT/Fe_2_O_3_ film sensor for ppb-level H_2_S detection. ACS Sens. **9**(10), 5197–5205 (2024). <https://doi.org/10.1021/acssensors.4c01342>
10. S. Kundu, G. Gorthala, R. Ghosh, Room temperature detection of H_2_S by two dimensional WS_2_ based chemiresistive sensors. Sens. Actuat. B Chem. **416**, 136018 (2024). <https://doi.org/10.1016/j.snb.2024.136018>
11. C. Li, S. Ramadan, H. Kan, L. Wang, Light-activated room temperature surface acoustic wave H_2_S sensor based on Bi_2_S_3_ nanoribbons. Sensors **25**(4), (2025). <https://doi.org/10.3390/s25041122>
12. R.K. Jha, A. Nanda, A. Yadav, R. Sai, N. Bhat, 2D-MoSe_2_/0D-ZnO nanocomposite for improved H_2_S gas sensing in dry air ambience. J. Alloys Compd. **926**, 166825 (2022). <https://doi.org/10.1016/j.jallcom.2022.166825>
13. G.M. Hingangavkar, S.A. Kadam, Y.-R. Ma, M. Selvaraj, R.N. Mulik et al., On the behavior of MoS_2_-rGO nanocomposites for chemiresistive H_2_S detection at room temperature. Sens. Actuat. B Chem. **399**, 134843 (2024). <https://doi.org/10.1016/j.snb.2023.134843>
14. N. Sakhuja, R.K. Jha, R. Sai, N. Bhat, ZnO nanorods grown on WS_2_ nanosheets for chemiresistive H_2_S sensing. ACS Appl. Nano Mater. **5**(7), 9241–9251 (2022). <https://doi.org/10.1021/acsanm.2c01580>
15. S.M. Lee, Y.J. Kim, S.J. Park, W.S. Cheon, J. Kim et al., *In-situ* growth of 2D MOFs as a molecular sieving layer on SnS_2_Nanoflakes for realizing ultraselective H_2_S detection. Adv. Funct. Mater. **35**(12), 2417019 (2025). <https://doi.org/10.1002/adfm.202417019>
16. M. Li, S. University, W. Ye, S. University, et al., Lead-free halide double perovskite Cs_2_AgBiCl_6_ for H_2_S trace detection at room temperature. ACS Sens. **10**(3), 2224–2233 (2025). <https://doi.org/10.1021/acssensors.4c03532>
17. Y. Sun, W. Guo, B. Wang, Y. Hou, W. Wang et al., Construction of a Co_3_(HITP)_2_/Ti3C2 MXene heterojunction for selective sensing of H_2_S with high resistance to humidity. Sens. Actuat. B Chem. **450**, 139224 (2026). <https://doi.org/10.1016/j.snb.2025.139224>
18. K. Zhou, H. Yang, Z. Du, Y. Yang, C. Zhu et al., Ultrahigh selectivity H_2_S gas sensor based CsPbBr_3_ perovskites *via* Pb–S bonding interaction. ACS Sens. **10**(1), 517–525 (2025). <https://doi.org/10.1021/acssensors.4c02980>
